# A self-complementary macrocycle by a dual interaction system

**DOI:** 10.1038/s41467-022-33357-y

**Published:** 2022-09-26

**Authors:** Yuta Sawanaka, Masahiro Yamashina, Hiroyoshi Ohtsu, Shinji Toyota

**Affiliations:** Department of Chemistry, School of Science, Tokyo Institute of Technology, Tokyo, Japan

**Keywords:** Self-assembly, Crystal engineering

## Abstract

Self-complementary assembly is one of the most promising phenomena for the formation of discrete assemblies, e.g., proteins and capsids. However, self-complementary assembly based on multiple host-guest systems has been scarcely reported due to the difficulty in controlling each assembly. Herein, we report a dual interaction system in which the key assembly direction is well regulated by both π-π stacking and hydrogen bonding to construct a self-complementary macrocycle. Continuous host-guest behavior of anthracene-based molecular tweezers during crystallization leads to successful construction of a cyclic hexamer, which is reminiscent of Kekulé’s monkey model. Furthermore, the cyclic hexamer in a tight and triple-layered fashion shows hierarchical assembly into cuboctahedron and rhombohedral assemblies in the presence of trifluoroacetic acid. Our findings would be potentially one of metal-free strategies for constructing anthracene-based supramolecular assemblies with higher-order structure.

## Abstract (web summary)

In nature, HIV capsid consists of single class of protein unit by self-complementarity. Here, the authors find that a molecular tweezer forms a cyclic hexamer by its continuous host-guest behavior, and constructs a large cuboctahedron by hierarchical assembly.

## Introduction

**Fig. 1:**
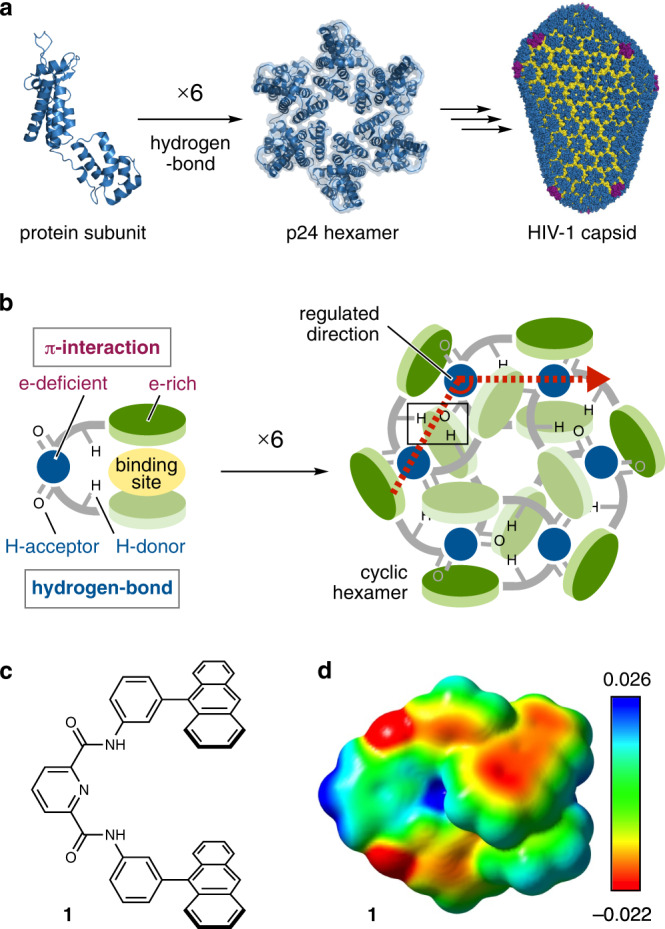
Schematic representations of self-complementary assembly. **a** Six protein subunits assembled through multiple hydrogen bonds to form a p24 protein hexamer of the HIV capsid. **b** Schematic representation of the dual interaction system for the construction of a self-complementary macrocycle in this work. **c** Chemical structure of anthracene-based molecular tweezers **1** and **d** its ESP map calculated by the density functional theory (DFT) method at the B3LYP-D3/6-31 G(d,p) level.

Ingenious molecules incorporating effective noncovalent bonding sites can form well-defined self-assemblies from a single class of molecules using their intrinsic properties. Such assemblies are often called “self-complementary assemblies”^1^, which are frequently shown in nature^2^. For example, the HIV-1 capsid is composed of the p24 protein hexamer, which is self-assembled by six protein subunits through multiple hydrogen bonds (Fig. 1a)^3,4^. In supramolecular chemistry, abundant self-complementary assemblies have been reported on the basis of intrinsic hydrogen bonds^5–8^, π-interactions^9–12^, and coordination bonds^13,14^. However, although host-guest behavior is a combination of multiple noncovalent interactions^6^ and ubiquitous phenomena in the field of science^15^, the construction of precise assemblies based on homologous host-guest systems is still a formidable challenge. This difficulty mainly arises for three reasons: (i) monomer design – both “host” and “guest” parts with high binding affinity have to be involved in a monomer molecule; (ii) binding mode – to progress consecutive bindings, the host-guest reactions should be carried out in an enclathration fashion, in which a host molecule partially binds to a guest^16^, rather than in a full encapsulation fashion; (iii) assembly direction – each host-guest complex should have sufficient rigidity since structural flexibility (i.e., less directionality of assembly) leads to the formation of infinite assemblies. Due to these limitations, the number of discrete self-complementary assemblies realized by host-guest reactions is overwhelmingly low even in noncovalent bond^17^ and heterologous systems thus far^18^.

To demonstrate the construction of a discrete self-complementary assembly^1^, we herein focused on a tweezer-like molecule (i.e., molecular tweezers), in which two aromatic panels are connected by a linker unit^19,20^. Pyridinedicarboxamide (PDA) is often incorporated into various functional molecules since intramolecular hydrogen bonding regulates two substituents on N atoms into a *syn* form^21,22^. According to recent achievements by Sessler’s and Haino’s research groups, PDA-based molecular tweezers can form self-complementary complexes in both head-to-head and head-to-tail modes^23–25^. In particular, head-to-tail assembly tends to form linear supramolecular polymers through donor-acceptor promoted π-π interactions^26,27^. We thus envisaged that providing a “regulated direction” to head-to-tail assembly as a secondary interaction could enable construction of a closed-ring assembly by capturing the other, such as the “six monkey model” of Kekulé’s anecdote^28^. To implement both donor-acceptor promoted π-π interactions and hydrogen bonding in concert, namely, the “dual interaction system”, we employed PDA-based molecular tweezers with polycyclic aromatic panels (Fig. 1b, left). Electron-rich aromatic units prefer to bind an electron-deficient pyridine ring moiety^29^. Afterwards, the π-π interaction assists intermolecular hydrogen bonding between the inner NH (H-donor) and outer carbonyl (H-acceptor). As a consequence of dual interactions, the second tweezers would have an orientation with a regulated direction, leading to construction of a macrocycle via continuous host-guest behavior (Fig. 1b, right).

Here, we report anthracene-based molecular tweezers **1** with a PDA linker (Fig. 1c). The electron density of the anthracene panels is much higher than that of the pyridine moiety, as confirmed by the electrostatic potential (ESP) map (Fig. 1d). By employing the dual interaction strategy, a supramolecular macrocycle is exclusively and efficiently constructed in the solid state. X-ray and thermal analyses reveal that the macrocycle is sufficiently stable in the solid state. Moreover, a capsid-shaped very large assembly, in which 108 molecules are assembled into a cuboctahedron geometry, is obtained by further assembly of the macrocycles.

## Results

### Formation of a self-complementary dimer in a solution

Anthracene-based molecular tweezers **1** was synthesized by amidation of 2,6-pyridinedicarbonyl dichloride with 9-(3-aminophenyl)anthracene in 93% yield (Supplementary Figs. 1–6). In the concentration-dependent ^1^H NMR analysis in CDCl_3_, pyridyl signals *H*_a_ and *H*_b_ showed a significant upfield shift upon increasing the concentration of **1**. These chemical shift changes arose from the aromatic shielding effect from the anthracene panels. Plotting the changes in chemical shift versus the concentration of **1** provided a binding curve that perfectly fits the simple dimerization model (Supplementary Fig. 17, Supplementary Table 1). The self-association constant *K*_a_ at 293 K was determined to be 314 M^–1^. According to the van’t Hoff plot, Δ*H* and *T*Δ*S* were estimated to be –27.3 kJ/mol and –13.3 kJ/mol, respectively. The formation of (**1**)_2_ is thus an enthalpy-driven process. Interestingly, a theoretical study revealed that head-to-tail assembled dimer is more stable by 7.4 kJ/mol than head-to-head assembled dimer (Supplementary Fig. 18). The pyridine signals show the most intense upfield shift, indicating that tweezers **1** prefers to form head-to-tail dimer (**1**)_2_ in CDCl_3_.

### Formation of a self-complementary macrocycle in the solid state

**Fig. 2:**
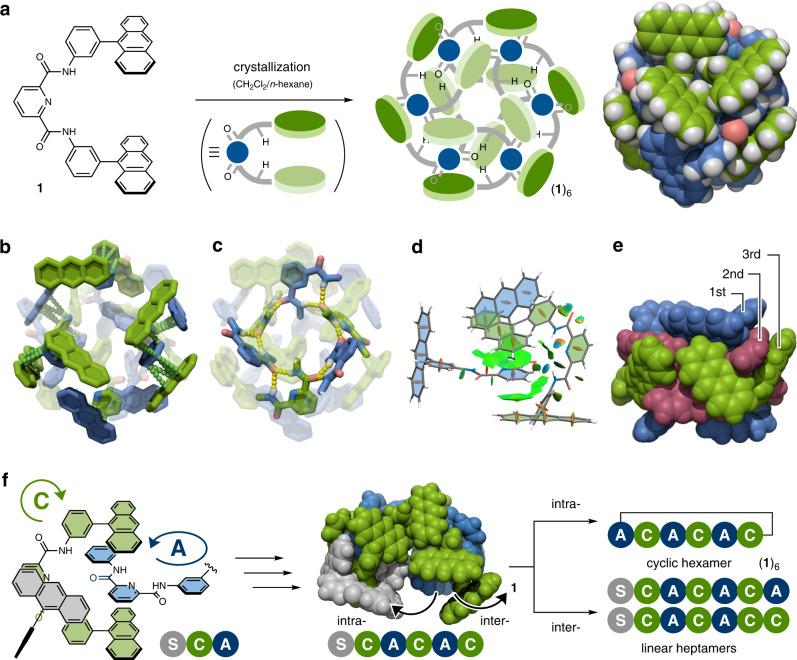
Formation of a self-complementary macrocycle. **a** Formation of self-complementary macrocycle (**1**)_6_, and its X-ray crystal structure. Highlighted interactions in (**1**)_6_: **b** π-π interactions (green dotted line) and **c** hydrogen bonding (yellow dotted line). **d** NCI plot calculated by the DFT method at the B3LYP-D3/6-31 G(d,p) level. A single unit of the head-to-tail complex was extracted for clarity (light green isosurface: π-π interaction; light blue isosurface: hydrogen bonding). **e** Representation of the triple-layered fashion of (**1**)_6_ (blue: 1st-layer; red: 2nd-layer; green: 3rd-layer). **f** Schematic representation of the assembling process of **1** to form cyclic hexamer (**1**)_6_ and linear heptamers.

In contrast, tweezers **1** formed the head-to-tail hexamer in the solid state. The slow diffusion of *n*-hexane into a CH_2_Cl_2_ solution of **1** for 1 day afforded colorless crystals with the triclinic *P*-1 space group. Single-crystal X-ray analysis unambiguously revealed that six independent tweezers molecules of **1** engaged each other to provide the macrocycle (**1**)_6_ of *S*_6_ symmetry (Fig. 2a, Supplementary Figs. 19-20). The hexagonal assembly (**1**)_6_ is ca. 22 Å in width and ca. 15 Å in height. The pyridine moiety of one molecule is intercalated by the two anthracene units of another molecule via a π-π interaction (ca. 3.4 Å for the C^…^C distance, Fig. 2b). Twelve sets of global intermolecular NH^…^OC hydrogen bonds are observed between one of the two carbonyl groups and the amide protons (ca. 2.2 Å, Fig. 2c). The noncovalent interaction (NCI) plot^30^ also supported the presence of these interactions (Fig. 2d). As a consequence of intermolecular hydrogen bonding, the direction of bound **1** was regulated at ca. 90° (Supplementary Fig. 21a), which is relatively smaller than the value of 120° for the ideal hexagonal shape. However, since the PDA linker and the anthracenes are orthogonal to each other, the six tweezers consecutively assemble in a zigzag pattern with 90° twisting, resulting in a chair-like cyclohexane conformation (Supplementary Fig. 21a). This assembling style may stabilize the hexameric structure, which is constructed by the cooperation of multiple noncovalent bonds. To avoid steric hindrance, both 3-(9-anthryl)phenyl moieties in **1** were twisted; their C(carbonyl)–N(amide)–C(phenyl)–C(phenyl) dihedral angles were –100.1° and +169.5° (Supplementary Fig. 21b). Although the formation of (**1**)_6_ by a dual interaction system can be deemed “crystallization-driven self-assembly”^31–38^, the resultant assembly based on the dual interaction system is completely different from any previous examples, even among PDA-based coordination macrocycles^39,40^.

As shown in the crystal packing, (**1**)_6_ exists as an entirely independent assembly and stacks in a multilayered fashion. A few intermolecular π-π interactions among each hexamer (**1**)_6_ are found with disordered CH_2_Cl_2_ and *n*-hexane (Supplementary Figs. 22,23). Two sets of anthracene moieties in **1** are in different environments: one is located inside and the other is along the side of the assembly. Notably, (**1**)_6_ can be considered a triple-layered macrocycle (Fig. 2e). In particular, the three parts in **1** described in Supplementary Fig. 24 correspond to 1st, 2nd, and 3rd layers. All of the layers roughly configurate macrocycle-like appearances. A multilayered molecule has been greatly attractive as a synthetic target^10,41–44^, but to the best of our knowledge, a macrocycle self-assembled in a triple-layered fashion has not been reported thus far. In the fluorescence spectra, whereas **1** showed a weak emission (*Φ*_F_ = 0.08) in a chloroform solution, (**1**)_6_ showed a relatively intense emission (*Φ*_F_ = 0.21) in the solid state owing to the suppression of thermal deactivation by the tight packing (Supplementary Fig. 25).

### Assembling process of the cyclic hexamer

The assembling process of **1** is remarkable. To construct cyclic hexamer (**1**)_6_, six sets of assembly processes are required. Tweezers **1** of *C*_2v_ symmetry has two sets of hydrogen-donor and -acceptor units. To simplify the analysis, we defined the direction of bound **1** as “clockwise” (C) or “anticlockwise” (A) (Fig. 2f and Supplementary Figs. 26a, 27). In the 1st assembly with the initial tweezers (S), the binding site of captured **1** points clockwise (C) or anticlockwise (A) relative to the anthracene face of tweezers S, leading to the formation of SC and SA as achiral compounds (Fig. 2f and Supplementary Fig. 26b). For the 2nd assembly, the orientation of bound **1** was determined by looking from the top of the anthracene panel, with the hydrogen-bonded carbonyl at the bottom (Fig. 2f and Supplementary Fig. 26c). According to this rule, 32 kinds of diastereomeric linear heptamers (except for their enantiomers) are statistically formed after six sets of assemblies (Supplementary Fig. 26a). For instance, when all assembly processes occur in the “C” direction, the helical heptamer SCCCCCC is provided (Supplementary Fig. 28). In the case of cyclic (**1**)_6_, binding processes alternate from side to side, and the intramolecular sixth binding of SCACAC must occur between the sixth and initial tweezers S for ACACAC formation (Fig. 2f). On the other hand, the intermolecular binding of SCACAC affords linear heptamer SCACACA or SCACACC (Fig. 2f).

### Selective formation and thermal stability of the macrocycle

**Fig. 3:**
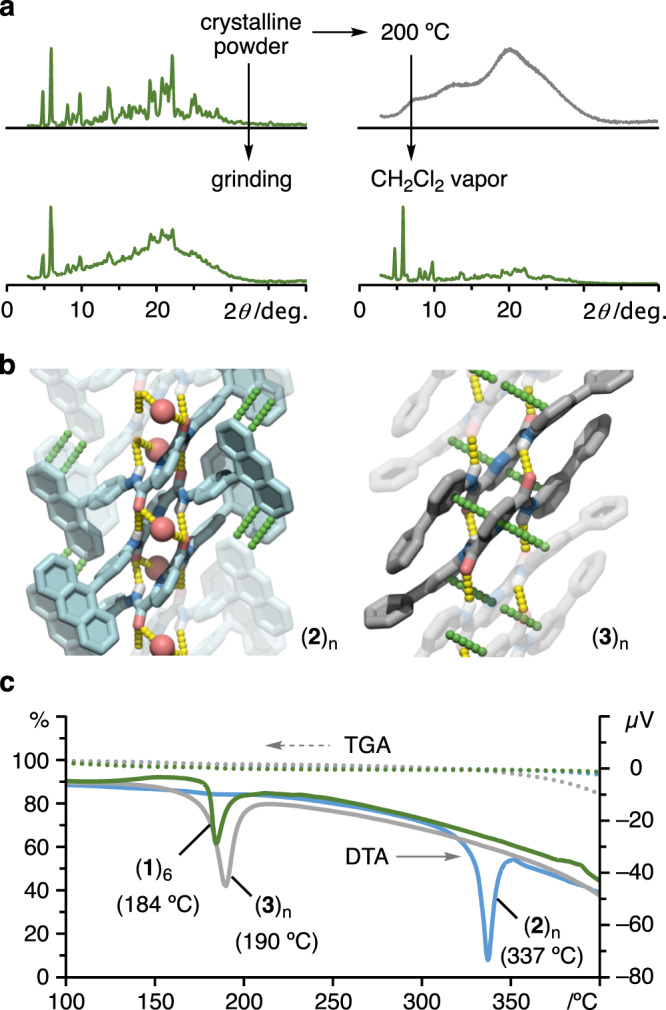
Structures and physical properties in the solid state. **a** PXRD patterns of the crystalline powders (**1**)_6_, after grinding, and the bulk amorphous powders after heating at 200 °C and then exposure to CH_2_Cl_2_ vapor (intensities are shown as arbitrary units). **b** Packing structures of **2** and **3** in the single crystals. Yellow and green dotted lines indicate hydrogen bonding and π-π interactions, respectively. Red balls in the (**2**)_n_ network are oxygen atoms of water molecules. **c** TG-DTA curves of (**1**)_6_, (**2**)_n_, and (**3**)_n_ from 100 to 400 °C (dotted line: TGA curve, solid line: DTA curve).

In the powder X-ray diffraction (PXRD) analysis of (**1**)_6_, the diffraction peaks are well-consistent with those of the simulated pattern (Fig. 3a (upper left), and Supplementary Fig. 30). The well-matched PXRD pattern implies that the crystalline powders have only structure consists of cyclic hexamer (**1**)_6_; no other structures like dimers nor linear oligomers. Due to the zigzag assembly, tweezers **1** cannot access the formation of odd-number cyclic oligomers (e.g., pentamer and heptamer). Steric hindrance and lack of structural rigidity might prevent the formation of tetramer and octamer, which are even-number cyclic oligomers (Supplementary Fig. 29). Furthermore, with the contribution of the crystal packing force, tweezers **1** exclusively forms the discrete cyclic hexamer (**1**)_6_ in the solid state from at least 32 kinds of linear oligomers. Cyclic hexamer (**1**)_6_ is relatively stable in the solid state. Although crystalline powders of (**1**)_6_ were ground in a mortar for 3 min, PXRD diffraction peaks derived from (**1**)_6_ were obviously found in the broad diffraction peak (Fig. 3a (lower left) and Supplementary Fig. 30). This result clearly revealed that the macrocyclic periodic structure remained in a large part of the structure while the other part becoming amorphous under mechanical stimulus. In contrast, cyclic hexamer (**1**)_6_ disassembled into an amorphous state once the crystalline powders melted at 200 °C. As shown in Fig. 3a (upper right) and Supplementary Fig. 31, all PXRD diffraction peaks disappeared, and diffraction of a broad nature indicating an amorphous state was observed. Interestingly, when the bulk amorphous powders of **1** were exposed to CH_2_Cl_2_ vapor for 2 h, the hexameric structure of **1** was regenerated, as confirmed by the PXRD analysis. All diffraction peaks are perfectly consistent with those of (**1**)_6_ (Fig. 3a (lower right) and Supplementary Fig. 31). The hexamer (**1**)_6_ was exclusively formed even in the presence of polyaromatic compounds (e.g., anthracene and pyrene) as competitors. Attenuated total reflection Fourier transform infrared (ATR-FTIR) spectroscopy showed that (**1**)_6_ exhibited two characteristic absorption bands due to amide C = O stretching at 1683 and 1656 cm^–1^, whereas amorphous **1** displayed a broad absorption at approximately 1685 cm^–1^ due to various conformations and associations (Supplementary Fig. 32).

In sharp contrast, both **2** and **3**, which have 4-(9-anthryl)phenyl and 3-biphenyl groups (Supplementary Figs. 7–16), respectively, as analogs of **1**, displayed columnar packing structures (**2**)_n_ and (**3**)_n_ through intermolecular 1D hydrogen bonds and π-interactions (Fig. 3b and Supplementary Figs. 33–36). Interestingly, the *m*-phenylene moieties of **3** inverted to form a linear structure rather than the tweezers form (Supplementary Fig. 35). As a consequence of these control experiments, we presumed that the 3-(9-anthryl)phenyl moieties play an important role in constructing discrete assemblies. The thermal stability of each assembly was evaluated by thermogravimetry-differential thermal analysis (TG-DTA) measurements over a range of 30 to 600 °C (Fig. 3c and Supplementary Fig. 37). The crystalline powders of (**1**)_6_ displayed a single endothermic peak at 184 °C, which was assigned to the melting point. On the other hand, although **2** is a constitutional isomer of **1**, (**2**)_n_ showed an endothermic peak at 337 °C, which is dramatically higher than that of (**1**)_6_. The observed high melting point arose from the robust intermolecular packing of **2**, to which infinite hydrogen bonds and complementary CH-π interactions among the 4 sets of anthracenes contributed (Supplementary Fig. 34). The crystal of (**3**)_n_ with a 1D hydrogen network exhibited an endothermic peak at 190 °C, the thermal stability of which was comparable to that of (**1**)_6_ (Fig. 3c).

### Hierarchical assembly of the macrocycle

**Fig. 4:**
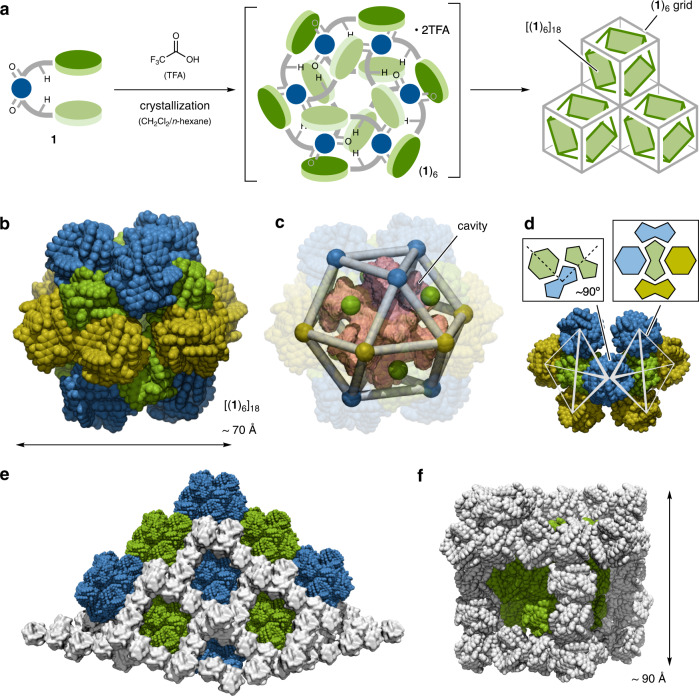
Hierarchical assembly based on (1)_6_ in the solid state. **a** Formation of [(**1**)_6_]_18_ and (**1**)_6_ grids by crystallization in the presence of TFA. **b**, **c** Extracted and schematic structures of [(**1**)_6_]_18_ with a cavity (pink color). To estimate the cavity, the relatively large holes on each surface of the cuboctahedron were obstructed by pseudoatoms with a 2.6 Å diameter. **d** Schematic representation of the assembly and intermolecular interactions of each hexamer (**1**)_6_. (**e**) Entire packing representation of trigonal crystal (**1**)_6_. All assemblies [(**1**)_6_]_18_ are encapsulated within the (**1**)_6_ grids in the crystal. Green and blue colors indicate [(**1**)_6_]_18_, and white color indicates (**1**)_6_ grids. Some grid hexamers (**1**)_6_ are omitted and displayed in surface representation for clarity. (**f**) Extracted set of [(**1**)_6_]_18_ (green color) and (**1**)_6_ (white color) grids.

Finally, we successfully constructed a further assembly based on cyclic hexamer (**1**)_6_. When crystals of **1** were grown from CH_2_Cl_2_/*n*-hexane in the presence of excess trifluoroacetic acid (TFA), trigonal-shaped pale-yellow crystals were obtained. According to X-ray analysis, surprisingly, cyclic hexamer (**1**)_6_ constructed a spherical assembly [(**1**)_6_]_18_ (Fig. 4a and Supplementary Fig. 38). The spherical [(**1**)_6_]_18_ resulted in an assembly of 18 sets of (**1**)_6_, which had a total of 108 molecules of **1** and was ca. 70 Å in diameter with a molecular weight of ca. 70 kDa (Fig. 4b and Supplementary Fig. 39a). The very large but relatively closed cavity within [(**1**)_6_]_18_ was calculated to be ~10000 Å^3^ by the MoloVol program (Fig. 4c and Supplementary Fig. 39b)^45^. Since cyclic hexamer (**1**)_6_ forms in a biconcave-lens fashion, (**1**)_6_ can interact with four other (**1**)_6_ molecules between the side and concave area in a cross shape through π-π interactions, leading to the formation of a square assembly (Fig. 4d). The distances between the centroids of each side are 24.5 and 25.1 Å. Nearby square assemblies share one (**1**)_6_ as a vertex with a 90° dihedral angle; consequently, [(**1**)_6_]_18_ forms a nearly complete cuboctahedron geometry. Notably, these phenomena from **1** towards [(**1**)_6_]_18_ via (**1**)_6_ can be considered hierarchical assembly^46,47^, which is reminiscent of the construction process of virus (e.g., HIV-1) capsids in nature^2,4^. Two molecules of TFA as an additive were found at the diagonal position of (**1**)_6_ with hydrogen bonds and halogen-π interactions^48^ among hexamers, which may be induced to form a trigonal packing structure (Supplementary Fig. 40). The emission of (**1**)_6_ was significantly quenched, probably due to charge-transfer between anthracene and electron deficient pyridyl-TFA complex^49^. Furthermore, all assemblies [(**1**)_6_]_18_ independently existed and were separated by the rhombohedral grid [(**1**)_6_]_n_, which was also formed by (**1**)_6_ in the crystal (Fig. 4e and Supplementary Figs. 41-42). The approximately 90 Å side of the unit grid is capable of encapsulating the [(**1**)_6_]_18_ assembly (Fig. 4f). All vertexes of cuboctahedron [(**1**)_6_]_18_ showed π-π interactions with grid sides and neighboring [(**1**)_6_]_18_ (Supplementary Fig. 43), which may contribute to stabilizing the giant assembly [(**1**)_6_]_18_.

## Discussion

We have successfully synthesized a crystalline-driven self-complementary macrocycle reminiscent of Kekulé’s monkey model based on consecutive host-guest complexations of molecular tweezers. The dual interaction system consisting of intermolecular π-π interactions and hydrogen bonds in concert provides exclusive formation of a cyclic hexamer in a triple-layered fashion from at least 32 types of linear isomers. The discrete and tight packing of the hexamer was well reflected in its fluorescent property and thermal stability. Furthermore, the cyclic hexamers transformed into both a giant spherical cuboctahedron composed of 108 molecular tweezers and rhombohedral grid assemblies upon the simple addition of TFA as a result of hierarchical assembly. Although self-complementary assemblies have been well studied using various types of molecules and intermolecular interactions, we show a self-complementary assembly based on the host-guest system. The present strategy involving two types of effective intermolecular interactions might not only be one of chemical tools for constructing metal-free assemblies, but also a way for assembling anthracenes in a circle, such as a light-harvesting antenna protein complex^50^.

## Methods

### General

All reagents and solvents were purchased from commercial sources and used as received. NMR spectra were recorded using a JEOL 500 MHz JNM-ECX500 spectrometer, a 500 MHz JNM-ECZ500 spectrometer, and a 400 MHz JNM-ECA400 spectrometer. Chemical shifts for ^1^H, ^13^C are reported in ppm on the *δ* scale; ^1^H and ^13^C signals were referenced to the residual solvent peak. Electrospray ionization time-of-flight (ESI-TOF) and high-resolution electrospray ionization mass spectrometry (HRMS-ESI) were performed on a Bruker micrOTOF II mass Spectrometer. Attenuated Total Reflection Fourier transform infrared (ATR-FTIR) spectra were measured by a JASCO FT/IR 4100 and a Shimadzu IRSpirit. Elemental analyses were performed on a Perkin-Elmer 2400 instrument. Single crystal X-ray crystallography was carried out with a Rigaku XtaLAB Synergy R/DW HyPix diffractometer with multi-layer mirror monochromated Cu Κ_α_ radiation (*λ* = 1.54184 Å) and processed on a CrysAlisPro 1.171 (Rigaku Oxford Diffraction, 2018). The structure was solved by direct methods using SHELXT-2014^51^ then refined and extended with SHELXL-2016^52^ using the OLex2.v1.2^53^ graphical user interfaces. Powder X-ray diffraction data were collected for samples on Si plate on a Rigaku Miniflex 600 instruments in house using reflection mode (Cu X-ray source with D/tex Ultra2 detector, 2 theta range: 3–50°, scan step: 0.01°). All PXRD samples were measured without pre-grinding. Thermal analysis was carried out with a Shimadzu DTG-60 instrument. UV-visible absorption spectroscopy was performed using a JASCO V-650 UV-Vis-NIR spectrophotometer. Samples were analyzed using a quartz cuvette with optical path lengths of 10 mm. Fluorescence spectroscopy was performed using a JASCO FP-8500 spectrofluorometer. Samples were analyzed at room temperature using quartz cuvettes with optical path lengths of 2.0 mm and 10 mm. Absolute fluorescence quantum yields were recorded on a Hamamatsu Quantaurus-QY C11347–01 with an integration sphere. All DFT calculations were performed by using Gaussian 16 program suite^54^. Molecular mechanics calculation was conducted by CONFLEX program (version 8, CONFLEX Corp., Japan)^55,56^ and Material Studio program (version 5.5.3, BIOVIA, USA). The cavity volume was calculated by MoloVol with 2.6 Å pseudoatoms (grid resolution: 0.5 Å).

### Synthesis of 1–3

2,6-Pyridinedicarboxylic acid (**A**; 3.34 g, 20.0 mmol) and dry-CH_2_Cl_2_ (20 mL) were added to a two-necked 100 mL flask under N_2_ atmosphere. Oxalyl dichloride (7.70 g, 5.2 mL, 60.6 mmol) and dry-DMF (10 µL) were sequentially added to the flask at room temperature. The resultant mixture was stirred for 24 h at room temperature, and then the solvent was evaporated under reduced pressure. The obtained solid was washed with *n*-hexane to give 2,6-pyridinedicarbonyl dichloride (**B**) as a white solid (3.59 g, 17.6 mmol, 88%). **B** (0.416 g, 2.04 mmol) was dissolved in dry-CH_2_Cl_2_ (80 mL) and then transferred into a 100 mL dropping funnel under N_2_. The solution was added dropwise to a solution of 9-(3-aminophenyl)anthracene (1.08 g, 4.01 mmol), triethylamine (2.0 mL), and dry-CH_2_Cl_2_ (8.0 mL) in a 300 mL two-necked flask over 3.5 h. The solution was stirred for 24 h at room temperature, and then the solvent was evaporated under reduced pressure. The crude mixture was dissolved in CHCl_3_ and then poured into water (100 mL). The organic phase was separated, and the aqueous phase was extracted with CHCl_3_ (50 mL×2). The combined organic extract was dried over Na_2_SO_4_, filtrated, and concentrated under reduced pressure. The obtained solid was washed with *n*-hexane to give **1** as a pale yellow solid (1.24 g, 91%). Under the same procedure, molecular tweezers **2** (81% yield) and **3** (99% yield) were prepared from 9-(4-aminophenyl)anthracene and 3-aminobiphenyl, respectively. Both single crystals of **2** and **3** were obtained from CH_2_Cl_2_/*n*-hexane at room temperature.

### For 1

^1^H NMR (500 MHz, CDCl_3_, 293 K, 20 mM) *δ* 7.05 (d, *J* = 7.4 Hz, 2H), 7.15 (t, *J* = 7.4 Hz, 4H), 7.28 (t, *J* = 8.0 Hz, 1H), 7.32 (t, *J* = 7.4 Hz, 2H), 7.40 (t, *J* = 7.4 Hz, 2H), 7.43-7.48 (m, 6H), 7.69 (d, *J* = 7.4 Hz, 2H), 7.86 (d, *J* = 8.0 Hz, 2H), 7.89 (d, *J* = 8.6 Hz, 4H), 8.29 (s, 2H), 9.10 (s, 2H). ^13^C NMR (125 MHz, CDCl_3_, 293 K, 20 mM) *δ* 119.6 (CH), 122.5 (CH), 124.9 (CH), 125.2 (CH), 125.5 (CH), 126.4 (CH), 126.7 (CH), 127.6 (CH), 128.4 (CH), 129.2 (CH), 129.8 (C_*q*_), 131.2 (C_*q*_), 135.9 (C_*q*_), 137.3 (C_*q*_), 137.9 (CH), 139.4 (C_*q*_), 147.5 (C_*q*_), 161.2 (C = O). HR MS (ESI-TOF, acetone): *m/z*: [*M* + Na]^+^ Calcd for C_47_H_31_N_3_O_2_Na, 692.2308; found 692.2327. FT-IR (amorphous, ATR, cm^–1^): 3321, 3053, 1685, 1605, 1583, 1531, 1488, 1431, 1403, 1359, 1306, 1164, 1141, 1074, 1013, 1000, 881, 841.

### For 2

^1^H NMR (500 MHz, CDCl_3_, 293 K, 10 mM) *δ* 7.37 (t, *J* = 7.5 Hz, 4H), 7.47 (t, *J* = 7.5 Hz, 4H), 7.54 (d, *J* = 8.0 Hz, 4H), 7.73 (d, *J* = 8.6 Hz, 4H), 8.01-8.10 (m, 8H), 8.27 (t, *J* = 8.0 Hz, 1H), 8.52 (s, 2H), 8.64 (d, *J* = 8.0 Hz, 2H), 9.75 (s, 2H). ^13^C NMR (125 MHz, CDCl_3_, 293 K, 10 mM) *δ* 120.3 (CH), 125.3 (CH), 125.6 (CH), 126.1 (CH), 126.8 (CH), 126.9 (CH), 128.6 (CH), 130.4 (C_*q*_), 131.5 (C_*q*_), 132.4 (CH), 135.7 (C_*q*_), 136.2 (C_*q*_), 136.6 (C_*q*_), 140.1 (CH), 149.2 (C_*q*_), 161.4 (C = O). Note: Due to the low solubility of **2** in CDCl_3_, the NMR spectra were measured at 10 mM. HR MS (ESI-TOF, acetone): *m/z*: [*M* + Na]^+^ calcd for C_47_H_31_N_3_O_2_Na, 692.2308; found 692.2315. Elemental analysis: Calcd for C_47_H_31_N_3_O_2_•(H_2_O)_1.3_•(CHCl_3_)_0.7_: C, 73.76; H, 4.45; N, 5.41. Found: C, 73.76; H, 4.20; N, 5.44.

### For 3

^1^H NMR (500 MHz, CDCl_3_, 293 K, 20 mM) *δ* 7.36 (t, *J* = 7.2 Hz, 2H), 7.40-7.44 (m, 6H), 7.46 (t, *J* = 7.8 Hz, 2H), 7.60 (d, *J* = 7.2 Hz, 4H), 7.71 (d, *J* = 7.8 Hz, 2H), 8.01 (s, 2H), 8.10 (t, *J* = 7.7 Hz, 1H), 8.48 (d, *J* = 7.7 Hz, 2H), 9.61 (s, 2H). ^13^C NMR (125 MHz, CDCl_3_, 293 K, 20 mM) *δ* 119.3 (CH), 119.4 (CH), 124.0 (CH), 125.8 (CH), 127.3 (CH), 127.8 (CH), 129.0 (CH), 129.7 (CH), 137.6 (C_*q*_), 139.7 (CH), 140.6 (C_*q*_), 142.5 (C_*q*_), 149.1 (C_*q*_), 161.5 (C = O). HR MS (ESI-TOF, acetone): *m/z*: [*M* + Na]^+^ calcd for C_31_H_23_N_3_O_2_Na, 492.1688; found 492.1676. Elemental analysis: Calcd for C_31_H_23_N_3_O_2_•(H_2_O)_0.2_: C, 78.69; H, 4.99; N, 8.88. Found: C, 78.73; H, 4.91; N, 8.70.

### Formation of self-complementary macrocycle (1)_6_

Recrystallization of **1** from CH_2_Cl_2_/*n*-hexane (2:1) at room temperature for 1 day gave (**1**)_6_ as a pale-yellow crystal. The formation of (**1**)_6_ was confirmed by NMR and both single crystal and powder X-ray analyses.

^13^C NMR (100 MHz, solid, 293 K) *δ* 163.0, 146.3, 143.0-114.5. FT-IR (ATR, cm^–1^): 3282, 3047, 1681, 1658, 1605, 1532, 1488, 1443, 1403, 1359, 1307, 1221, 1165, 1141, 1077, 1013, 1002, 953, 882, 841. Elemental analysis: Calcd for (C_47_H_31_N_3_O_2_)_6_•(CH_2_Cl_2_)_3.3_•(C_6_H_14_)_0.3_: C, 79.73; H, 4.59; N, 5.83. Found: C, 79.68; H, 4.59; N, 5.85.

### Formation of hierarchical assemblies based on (1)_6_

Recrystallization of **1** from CH_2_Cl_2_/*n*-hexane (2:1) in the presence of trifluoroacetic acid (excess) at room temperature for 1 day gave (**1**)_6_•(TFA)_2_ as a pale-yellow crystal. The formation of (**1**)_6_•(TFA)_2_ was confirmed by single crystal X-ray analysis.

FT-IR (ATR, cm^–1^): 3285, 3049, 2855, 2701, 2541, 1785, 1653, 1607, 1558, 1534, 1405, 1358, 1308, 1212, 1159, 1077, 1014, 1001, 884, 843, 790, 734, 702, 683, 595, 585, 554, 533, 425, 420. Elemental analysis: Calcd for (C_47_H_31_N_3_O_2_)_6_•(CF_3_COOH)_2_•(CH_2_Cl_2_)_3.5_•(C_6_H_12_)_0.2_: C, 76.55; H, 4.37; N, 5.53. Found: C, 76.55; H, 4.20; N, 5.51.

## Supplementary information

Supplementary Information

## Data Availability

The authors declare that the data supporting the findings of this study are available within the Supplementary Information files and from the corresponding author upon request. CCDC 2158897, 2158909, 2158891, and 2158899 contain the supplementary crystallographic data for the structure reported in this article. The data can be obtained free of charge from The Cambridge Crystallographic Data Centre (CCDC) via www.ccdccam.ac.uk/data_request/cif.
