# Supplementary material for: A self-complementary macrocycle by a dual interaction system
Source: Nat Commun. 2022 Sep 26;13:5648. doi: 10.1038/s41467-022-33357-y (PMC9512892; doi:10.1038/s41467-022-33357-y)
Supplement: Supplementary file 1 — Supplementary Information [file 41467_2022_33357_MOESM1_ESM.pdf]

Supplementary Information for

A Self-Complementary Macrocycle by a Dual Interaction System

Yuta Sawanaka[1], Masahiro Yamashina[1]*, Hiroyoshi Ohtsu[1], and Shinji Toyota[1]*

[1]Department of Chemistry, School of Science, Tokyo Institute of Technology, 2-12-1 Ookayama, Meguro-ku, Tokyo 152-8551, Japan.

*E-mail: yamashina@chem.titech.ac.jp, stoyota@chem.titech.ac.jp

Table of Contents

1. Supplementary Figures and Tables S3

1.1. Characterization of **1-3** S3

1.2. The dimerization constant and van't Hoff plot S11

1.3. The optimized structures of self-complementary dimer S12

1.4. Solid state ^{13}C NMR of (**1**)$_6$ S13

1.5. Single crystal X-ray diffraction of (**1**)$_6$ S14

1.6. A triple-layered macrocycle (**1**)$_6$ S17

1.7. UV-vis and fluorescence of (**1**)$_6$ S17

1.8. Assembling process of (**1**)$_6$ S18

1.9. Powder X-ray diffractions and IR measurement of (**1**)$_6$ S21

1.10. Single crystal X-ray diffraction of (**2**)$_n$ and (**3**)$_n$ S23

1.11. Thermal analysis S27

1.12. Single crystal X-ray diffraction of (**1**)$_6$•(TFA)$_2$ S28

2. Supplementary Methods S35

2.1. General procedure for the synthesis of aminophenyl precursors S35

2.2. Synthesis of **1-3** S36

2.3. Formation of self-complementary dimer S37

2.4. Formation of self-complementary macrocycle S37

2.5. Formation of hierarchical assemblies based on (**1**)$_6$ S38

3. Supplementary References S39

1. Supplementary Figures and Tables

1.1. Characterization of 1-3

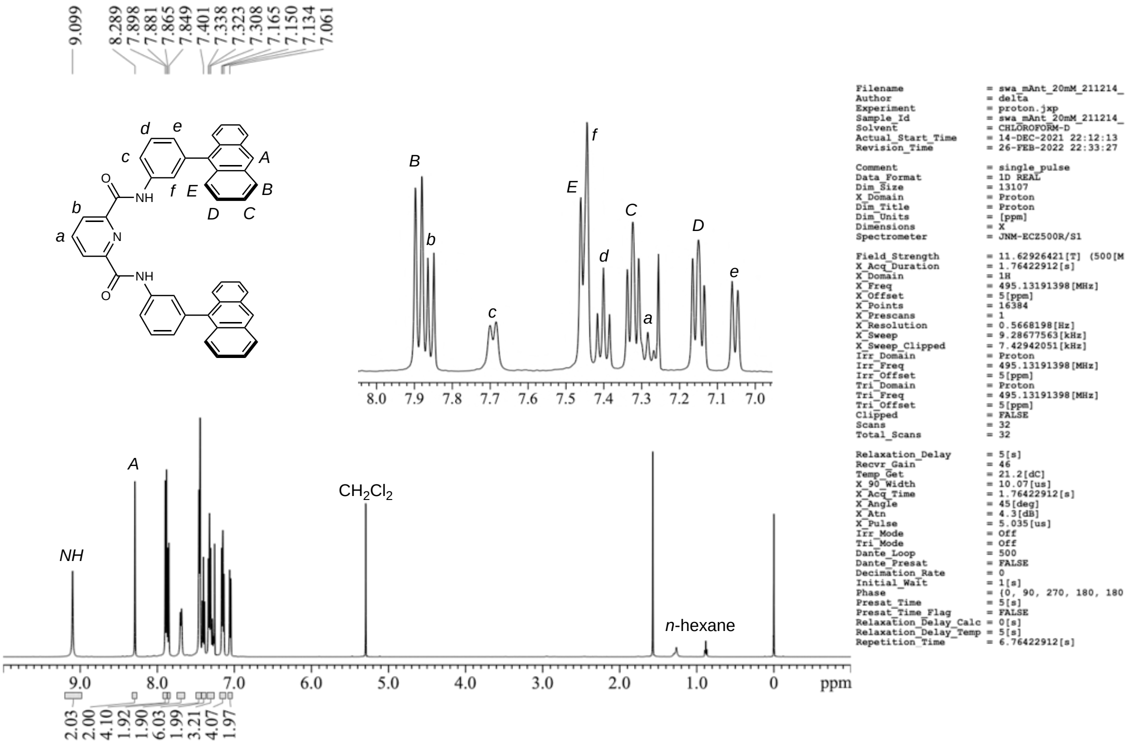

Supplementary Figure 1. ^{1}H NMR spectrum (500 MHz, $CDCl_3$, 293 K) of **1**.

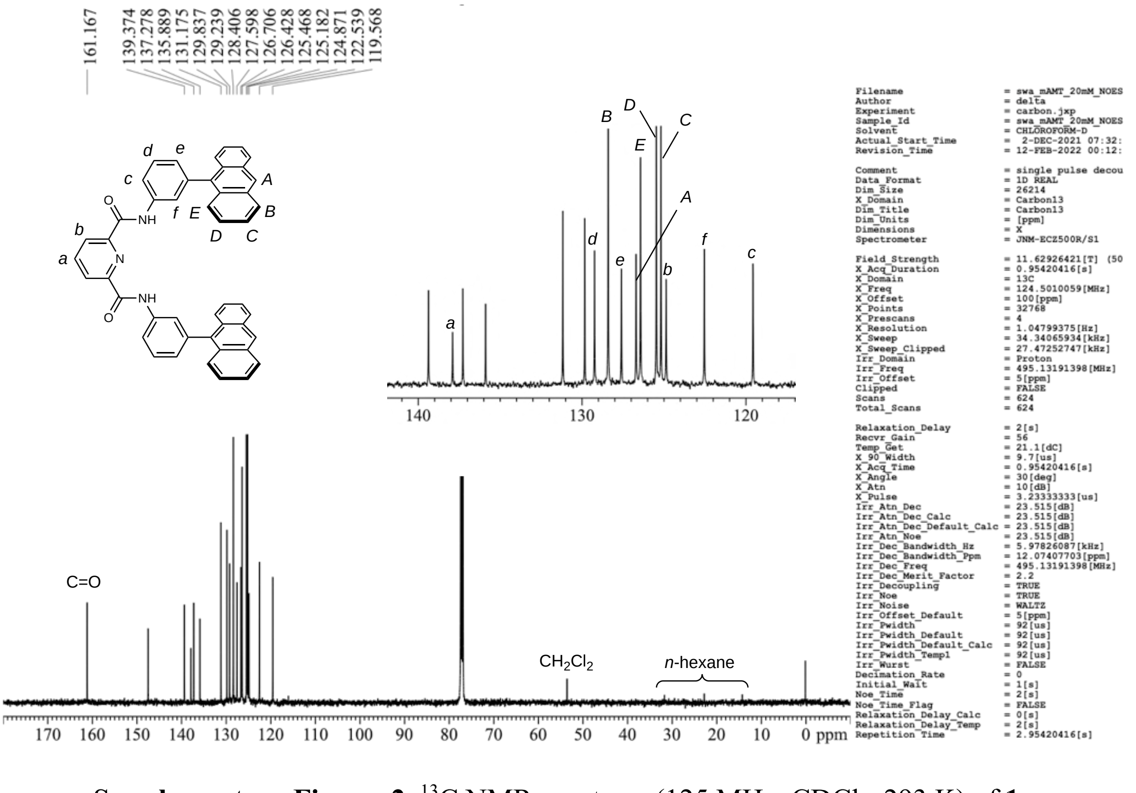

Supplementary Figure 2. ^{13}C NMR spectrum (125 MHz, $CDCl_3$, 293 K) of **1**.

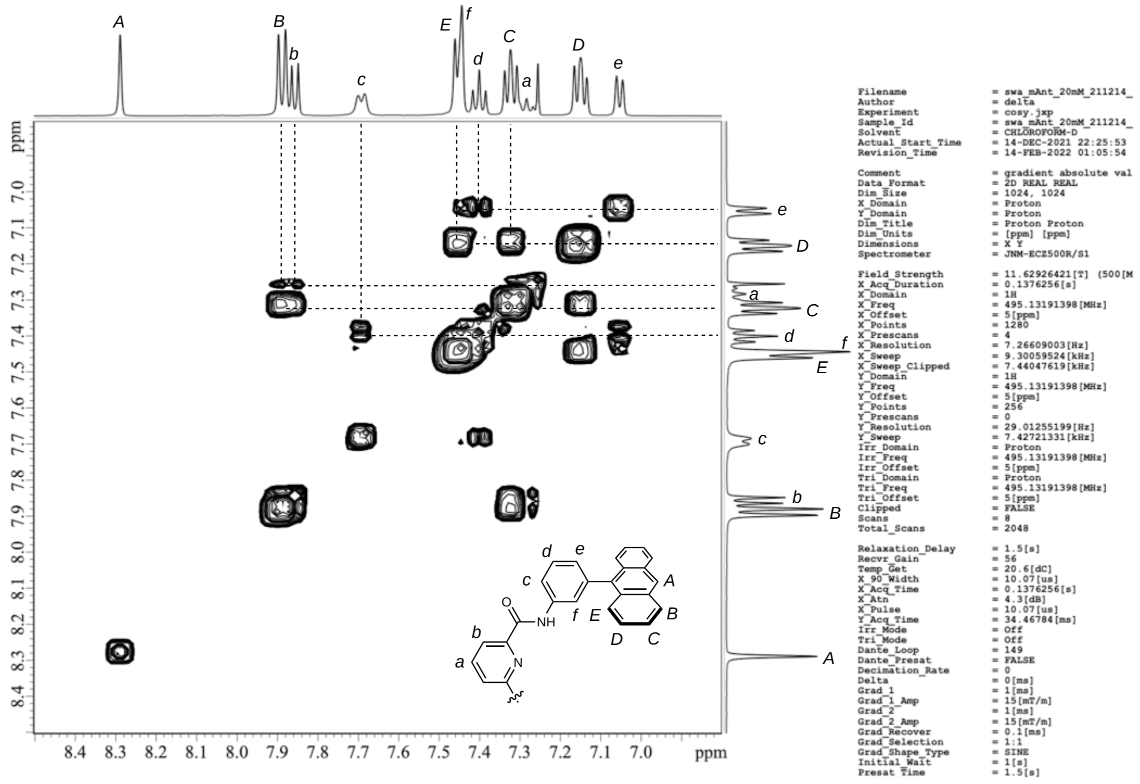

Supplementary Figure 3. ^{1}H-^{1}H COSY spectrum (500 MHz, $CDCl_3$, 293 K) of **1**.

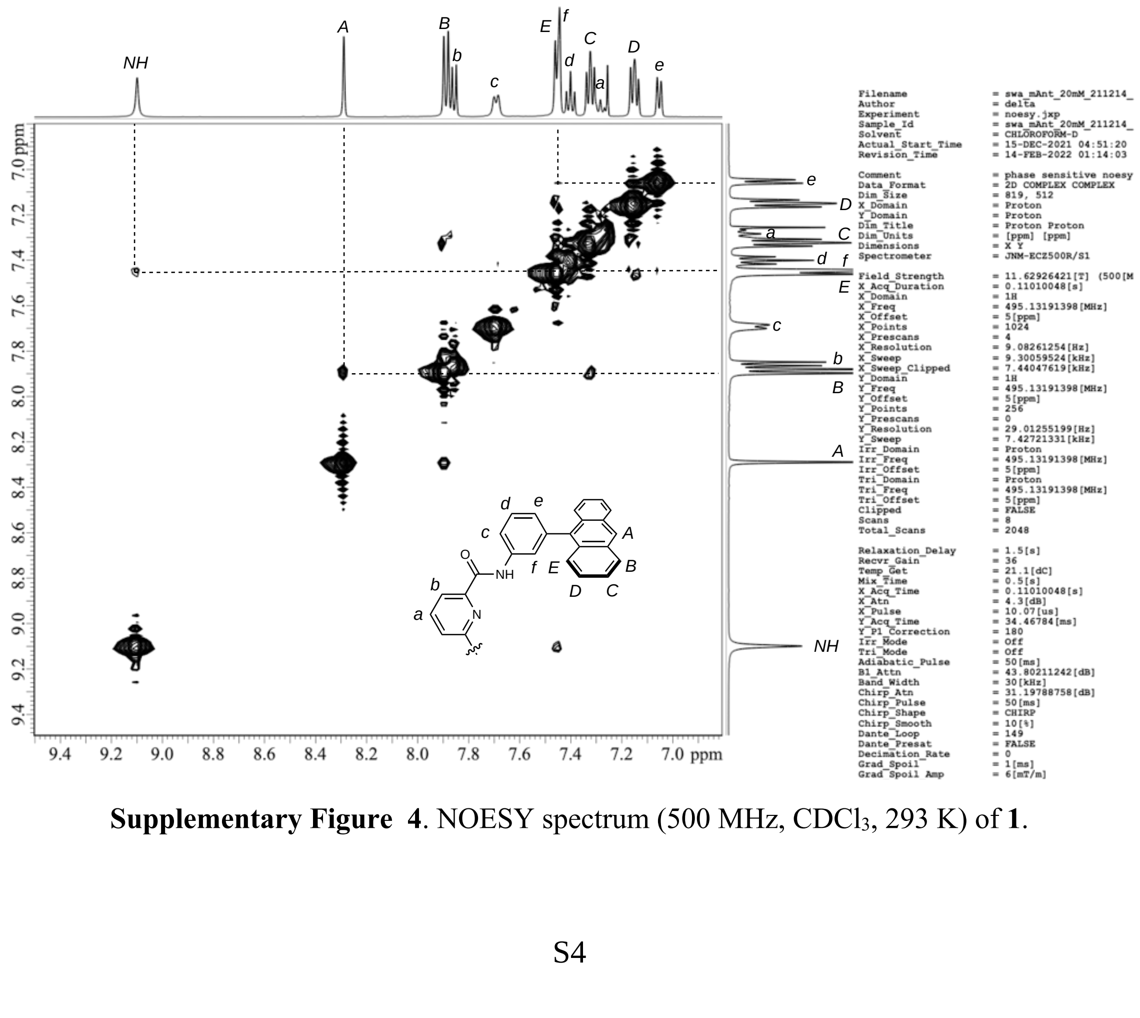

Supplementary Figure 4. NOESY spectrum (500 MHz, $CDCl_3$, 293 K) of **1**.

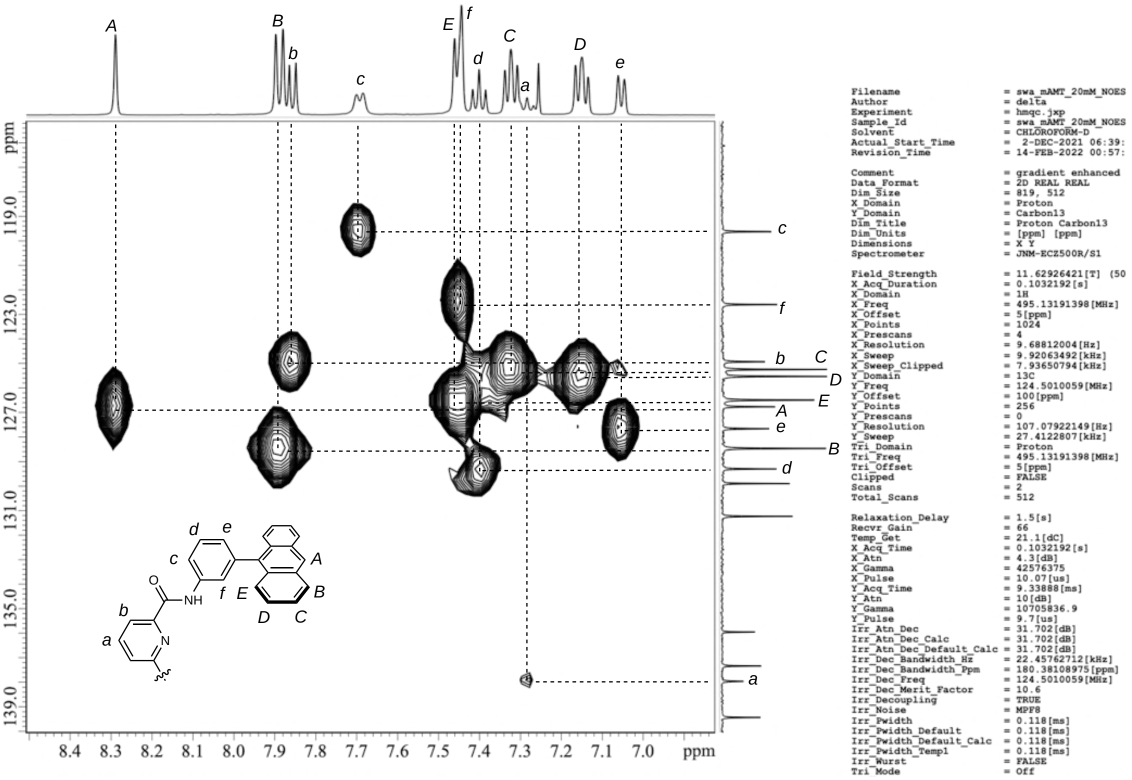

Supplementary Figure 5. HMQC spectrum (500 MHz, $CDCl_3$, 293 K) of **1**.

Display Report

Analysis Info

Acquisition Date 1/7/2022 1:48:31 PM

Analysis Name D:\Data\Toyota\YSawanaka\YS220106-1-000003.d

Method esi_posi_low.m

Operator BDAL@DE

Sample Name YS220106-1-

Instrument micrOTOF 213750.10321

Comment

Acquisition Parameter

Source Type	ESI	Ion Polarity	Positive	Set Nebulizer	0.3 Bar
Focus	Not active			Set Dry Heater	180 °C
Scan Begin	50 m/z	Set Capillary	4500 V	Set Dry Gas	4.0 l/min
Scan End	1600 m/z	Set End Plate Offset	-500 V	Set Divert Valve	Waste

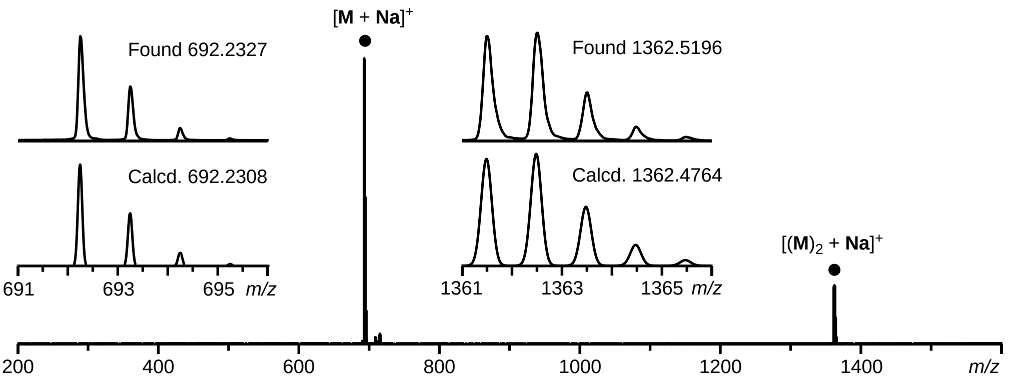

Supplementary Figure 6. HR MS (ESI-TOF) spectrum (acetone) of **1**.

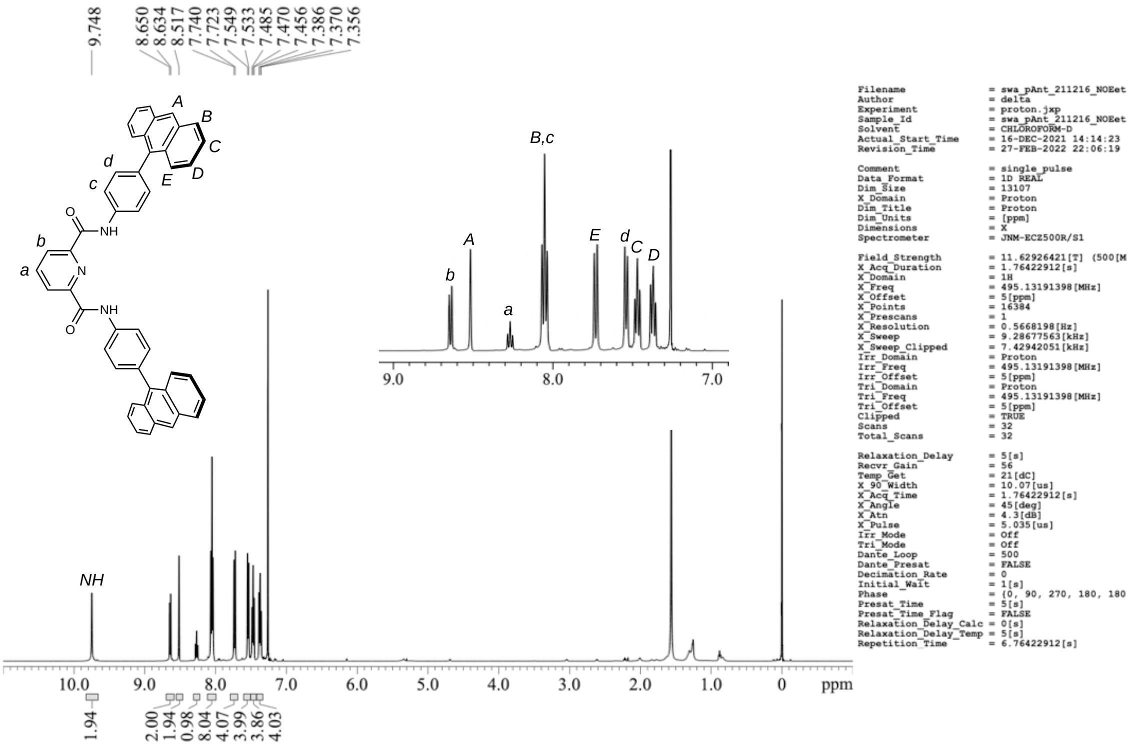

Supplementary Figure 7. ^{1}H NMR spectrum (500 MHz, $CDCl_3$, 293 K) of **2**.

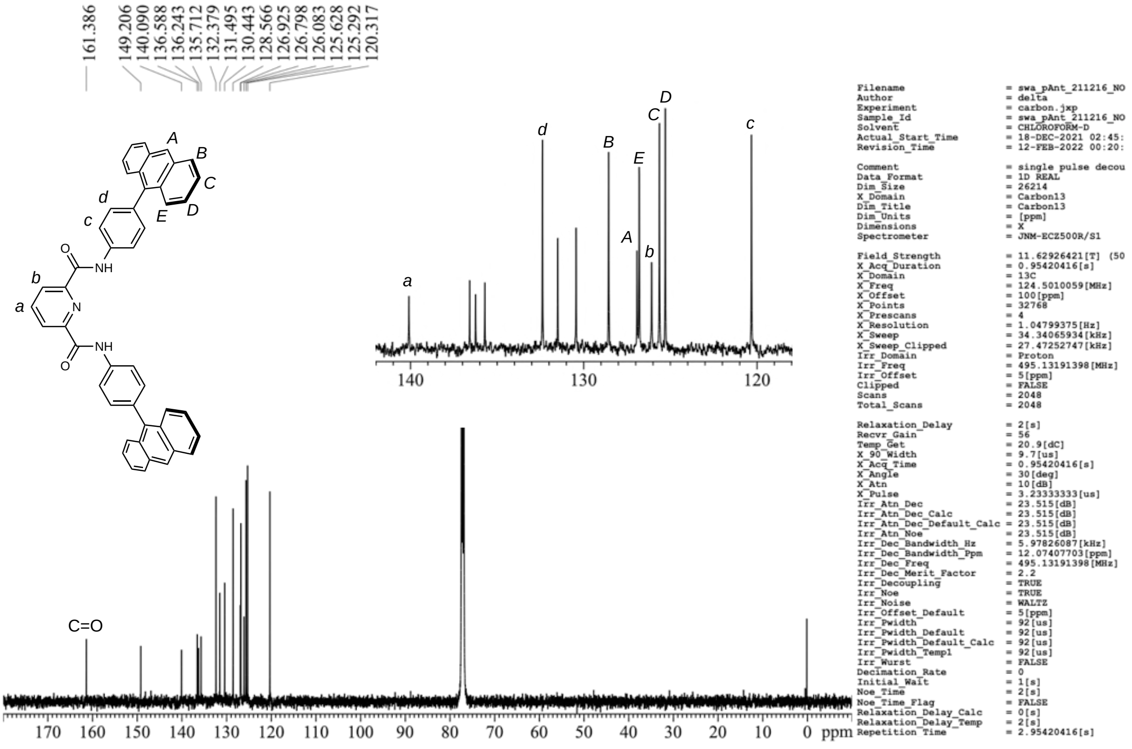

Supplementary Figure 8. ^{13}C NMR spectrum (125 MHz, $CDCl_3$, 293 K) of **2**.

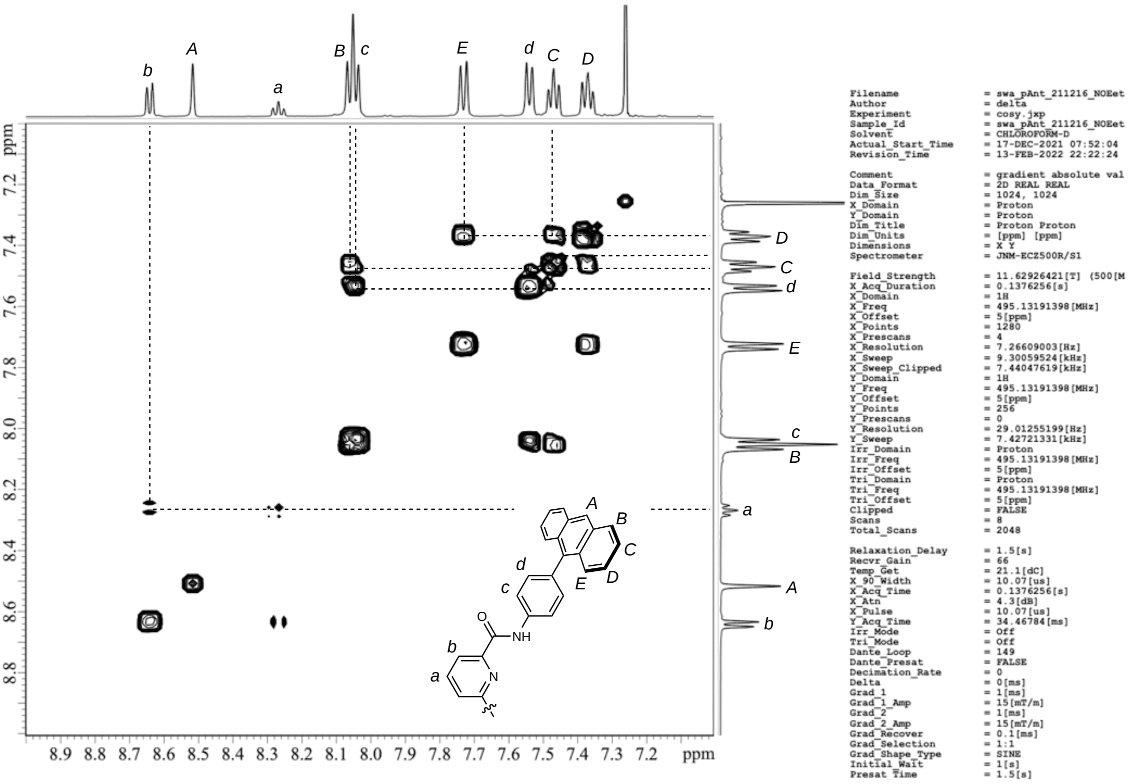

Supplementary Figure 9. ^{1}H-^{1}H COSY spectrum (500 MHz, $CDCl_3$, 293 K) of **2**.

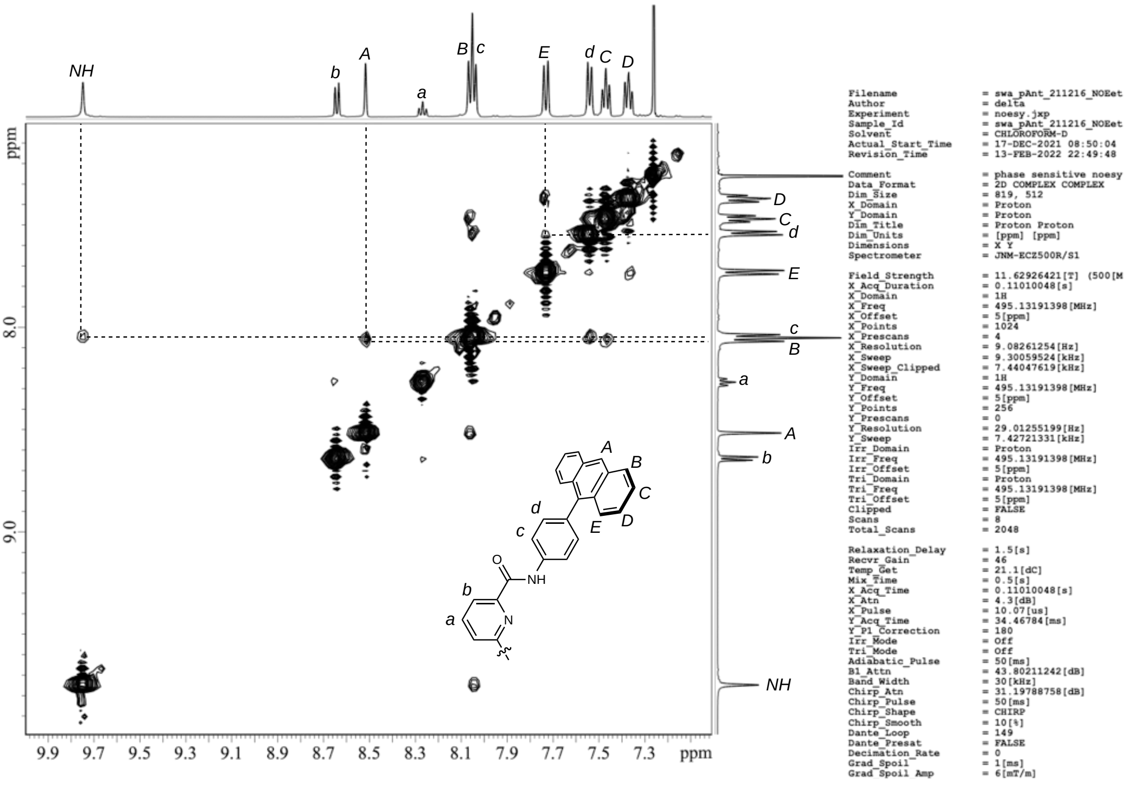

Supplementary Figure 10. NOESY spectrum (500 MHz, $CDCl_3$, 293 K) of **2**.

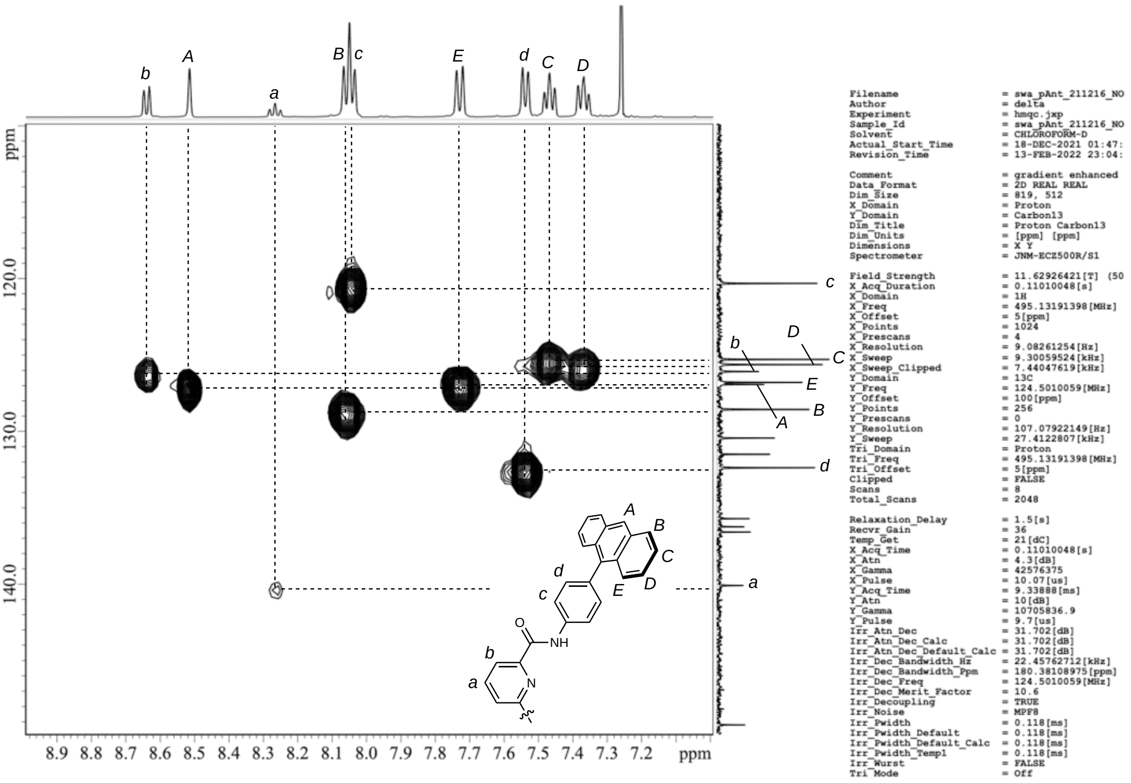

Supplementary Figure 11. HMQC spectrum (500 MHz, $CDCl_3$, 293 K) of **2**.

Display Report

Analysis Info

Analysis Name	D:\Data\Toyota\YSawanaka\YS220106-2-000002.d	Acquisition Date	1/7/2022 2:01:45 PM
Method	esi_posi_low.m	Operator	BDAL@DE
Sample Name	YS220106-2-	Instrument	micrOTOF 213750.10321
Comment			

Acquisition Parameter

Source Type	ESI	Ion Polarity	Positive	Set Nebulizer	0.3 Bar
Focus	Not active			Set Dry Heater	180 °C
Scan Begin	50 m/z	Set Capillary	4500 V	Set Dry Gas	4.0 l/min
Scan End	1600 m/z	Set End Plate Offset	-500 V	Set Divert Valve	Waste

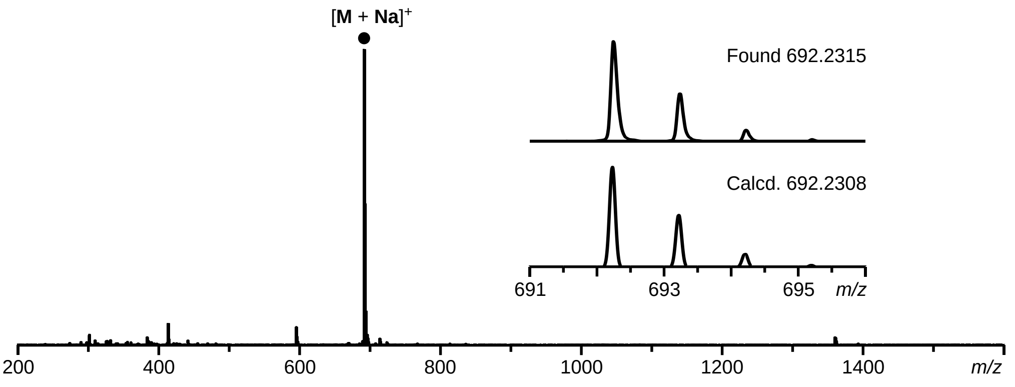

Supplementary Figure 12. HR MS (ESI-TOF) spectrum (acetone) of **2**.

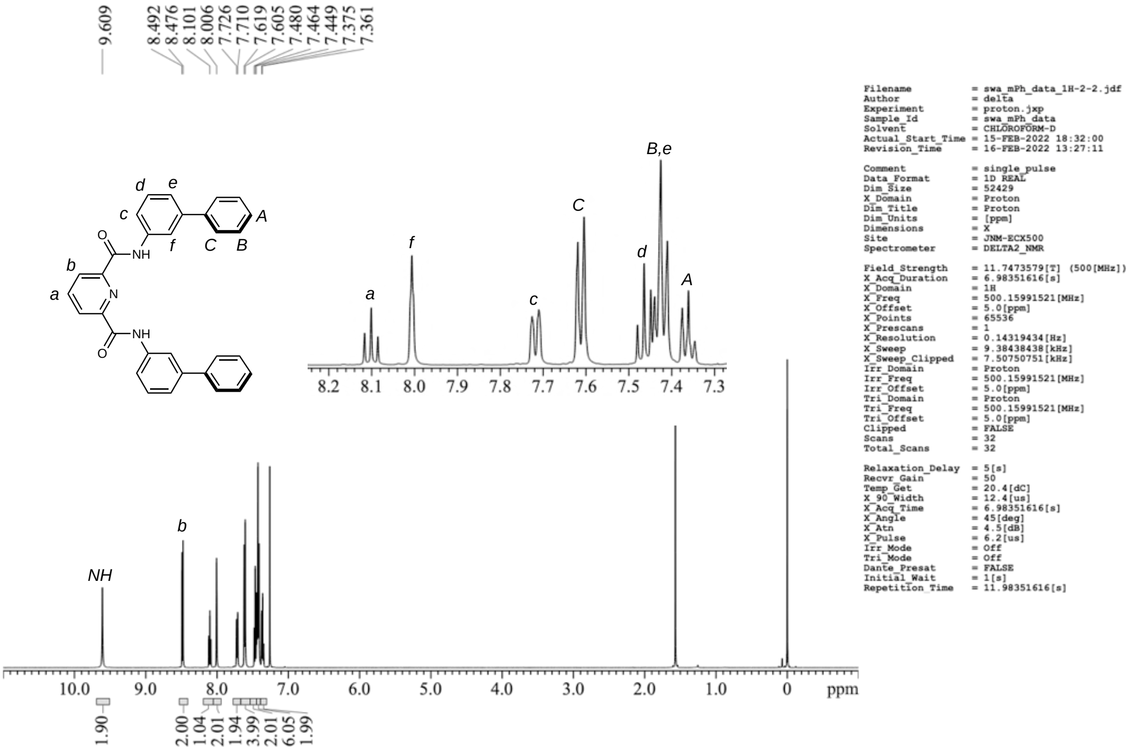

Supplementary Figure 13. ^{1}H NMR spectrum (500 MHz, $CDCl_3$, 293 K) of **3**.

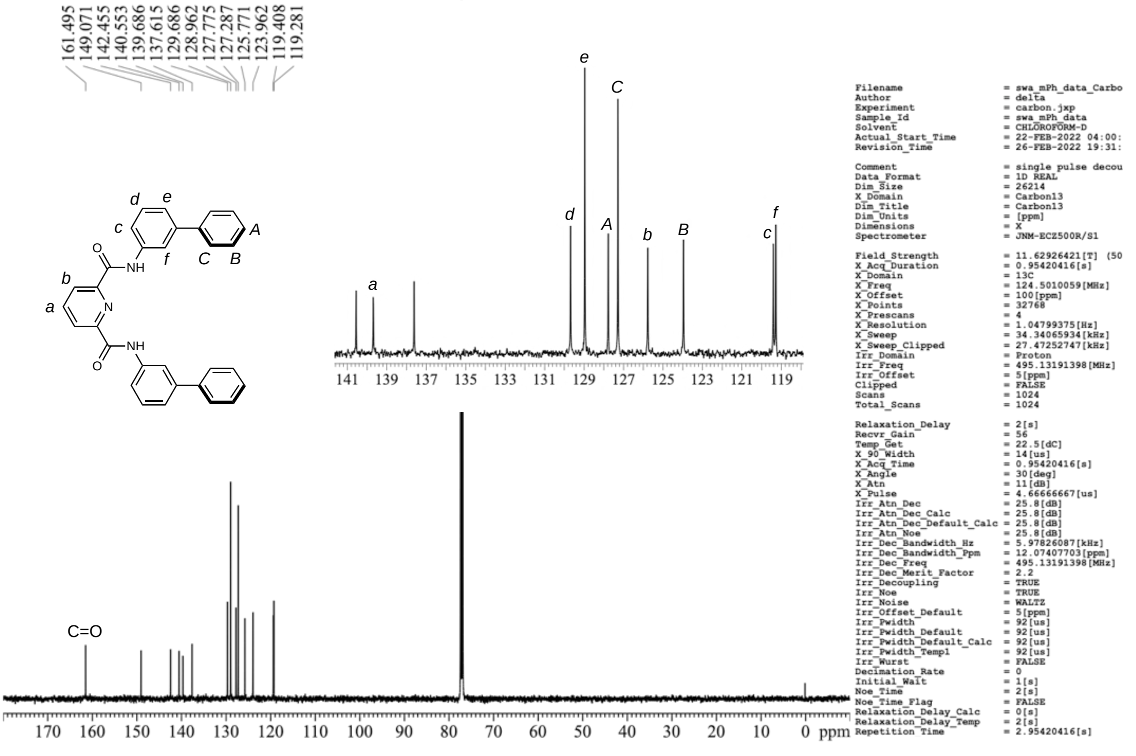

Supplementary Figure 14. ^{13}C NMR spectrum (125 MHz, $CDCl_3$, 293 K) of **3**.

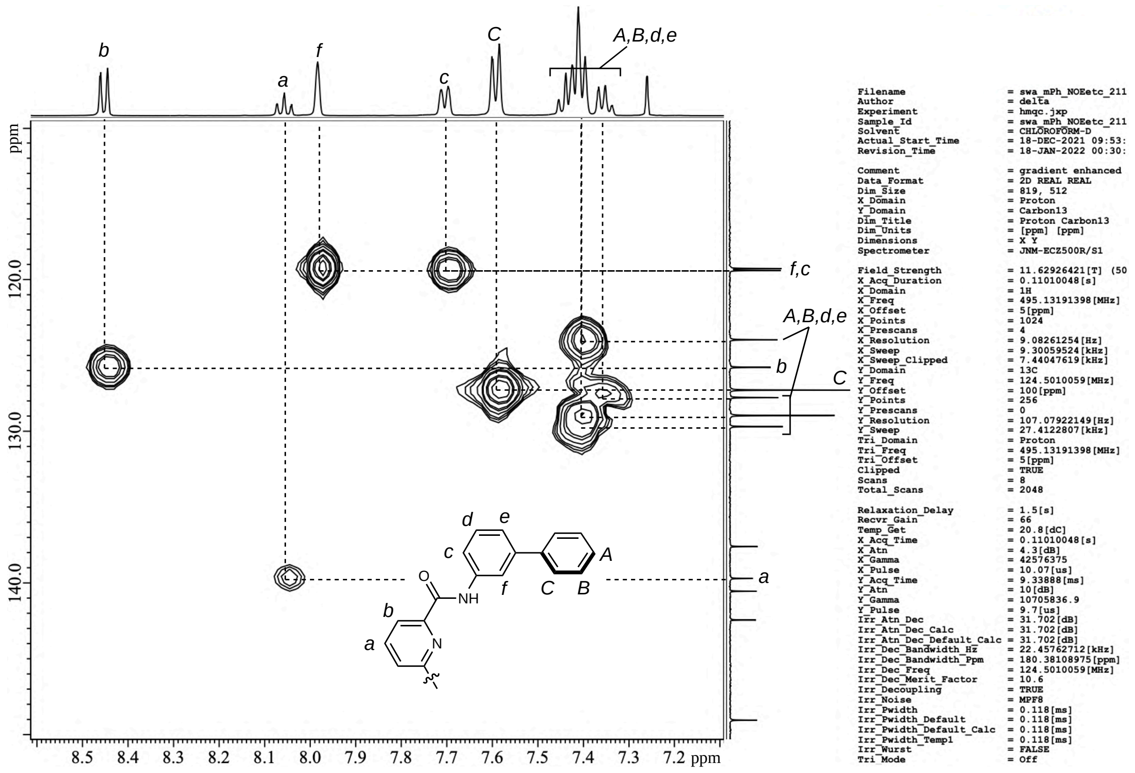

Supplementary Figure 15. HMQC spectrum (500 MHz, $CDCl_3$, 293 K) of **3**.

Display Report

Analysis Info

Analysis Name D:\Data\Toyota\YSawanaka\YS220106-3-000001.d
Method esi_posi_low.m
Sample Name YS220106-3-
Comment

Acquisition Date 1/7/2022 2:16:06 PM

Operator BDAL@DE
Instrument micrOTOF 213750.10321

Acquisition Parameter

Source Type	ESI	Ion Polarity	Positive	Set Nebulizer	0.3 Bar
Focus	Not active			Set Dry Heater	180 °C
Scan Begin	50 m/z	Set Capillary	4500 V	Set Dry Gas	4.0 l/min
Scan End	1600 m/z	Set End Plate Offset	-500 V	Set Divert Valve	Waste

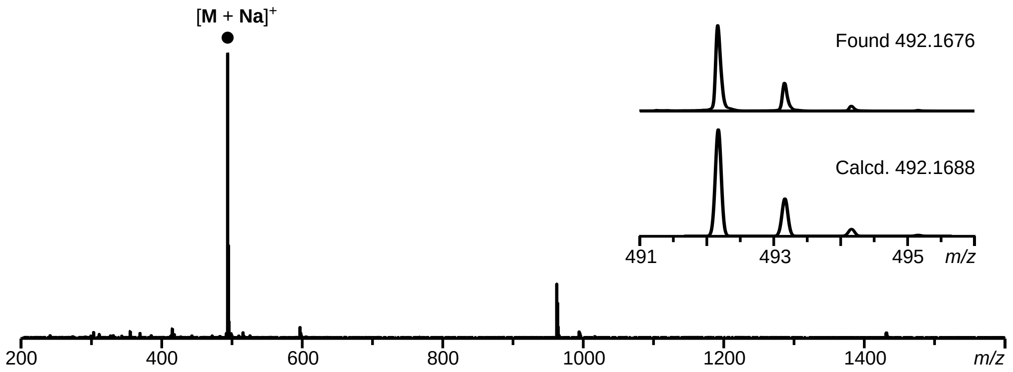

Supplementary Figure 16. HR MS (ESI-TOF) spectrum (acetone) of **3**.

1.2. The dimerization constant and van't Hoff plot

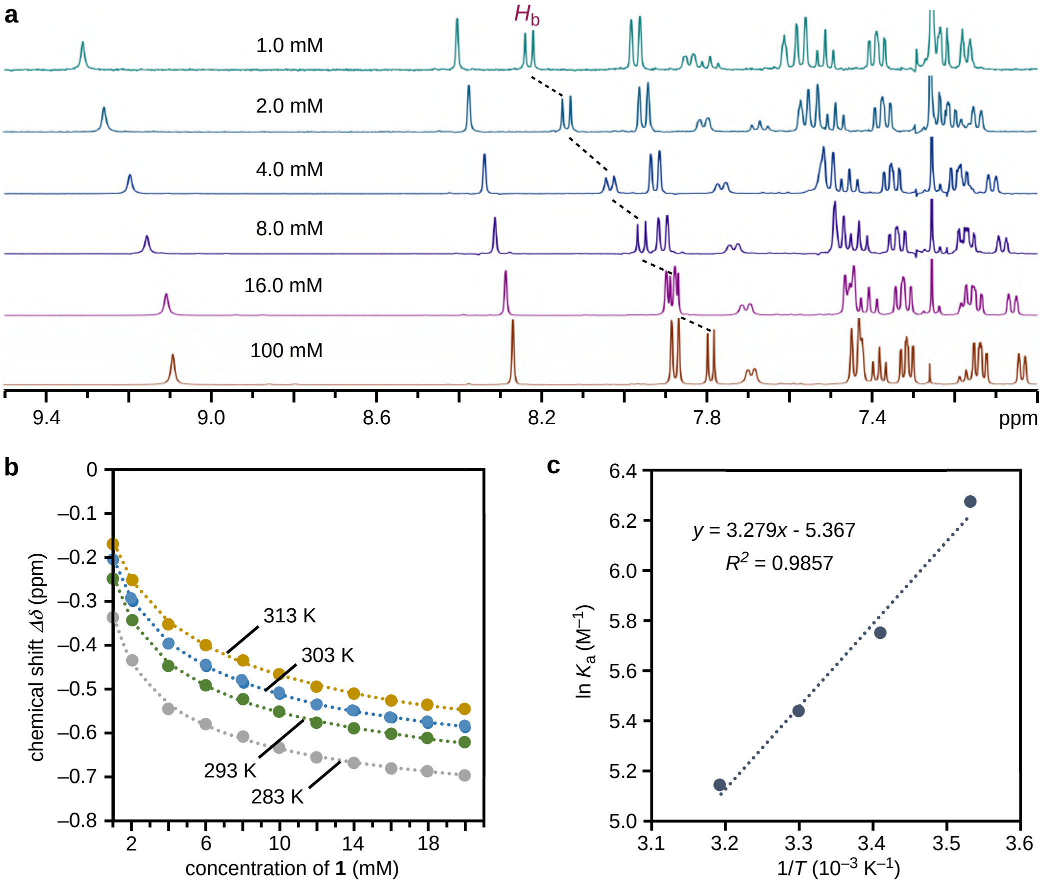

Supplementary Figure 17. Titration experiments. (a) Concentration-dependent ^{1}H NMR spectra (500 MHz, 293 K, $CDCl_3$) of **1**. (b) Temperature-dependent titration curves of **1** using H_b proton at 283, 293, 303, and 313 K, and its (c) van't Hoff plot.

Supplementary Table 1. Summary of titration. (a) Concentration-dependent ^{1}H NMR chemical shits of **1** at 283, 293, 303, and 313 K, and (b) thermodynamic parameters for the dimerization of **1**.

a

[**1**] (mM)	283	293	303	313
	(K)			
1	8.196	8.251	8.299	8.339
2	8.100	8.157	8.210	8.257
4	7.990	8.054	8.106	8.155
6	7.955	8.008	8.059	8.107
8	7.928	7.977	8.025	8.073
10	7.902	7.950	7.997	8.043
12	7.879	7.925	7.970	8.013
14	7.867	7.912	7.956	7.999
16	7.855	7.898	7.941	7.983
18	7.848	7.890	7.932	7.974
20	7.839	7.881	7.922	7.964

b

T (K)	$1/T$ (10^{-3} K^{-1})	K_a* (M^{-1})	$\ln K_a$
283	3.53	530	6.27
293	3.41	314	5.75
303	3.30	230	5.44
313	3.19	172	5.15

*: Association constant (K_a) values for the monomer-dimer exchange model were calculated by curve fitting and a nonlinear least-squares method using the SOLVER program[4]

1.3. The optimized structures of self-complementary dimer

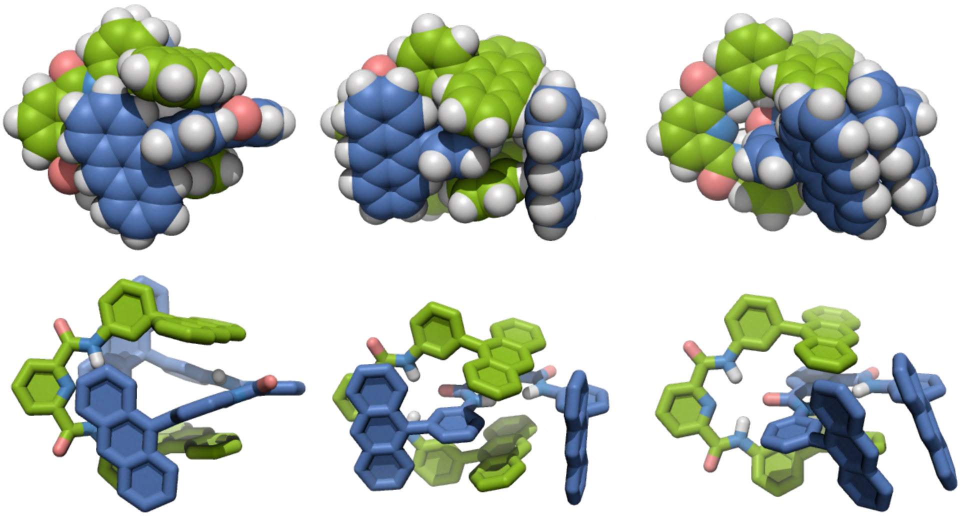

mode	head-to-head	head-to-tail	head-to-tail
ΔE (kJ mol^{-1})	0	–7.4	+0.35

Supplementary Figure 18. The optimized structures of three possible self-complementary dimers and their energy differences based on the head-to-head dimer calculated by CONFLEX and then DFT method at the B3LYP-D3/6-31G(d,p) level. Hydrogen atoms (except N*H*) were omitted for clarity.

1.4. Solid state ^{13}C NMR of (1)$_6$

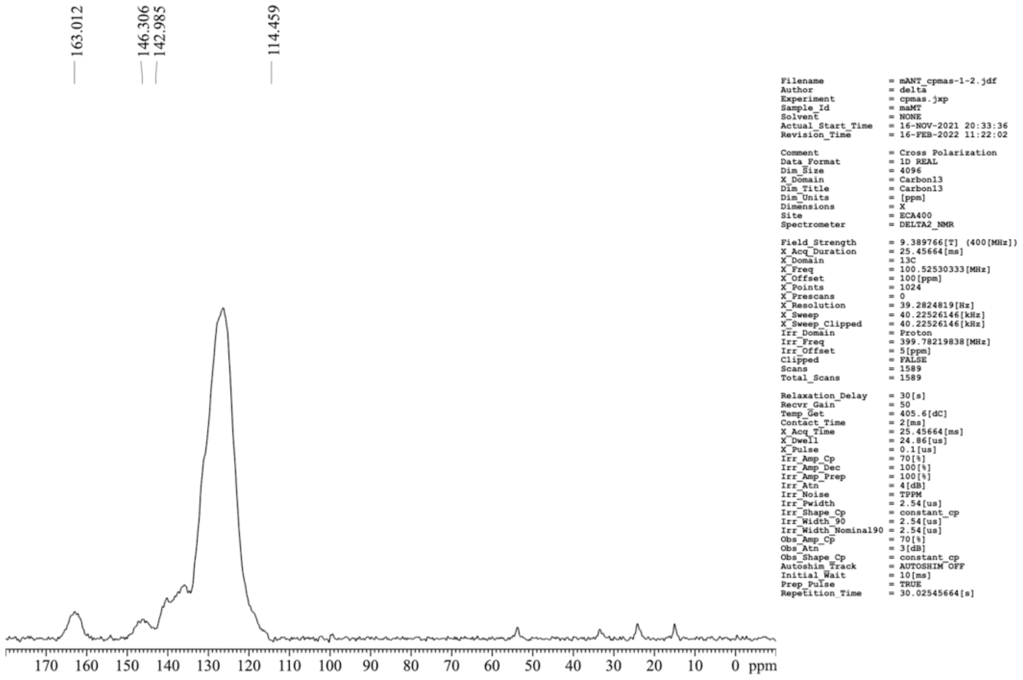

Supplementary Figure 19. ^{13}C NMR spectrum (100 MHz, solid, 293 K) of (**1**)$_6$.

1.5. Single crystal X-ray diffraction of (1)$_6$

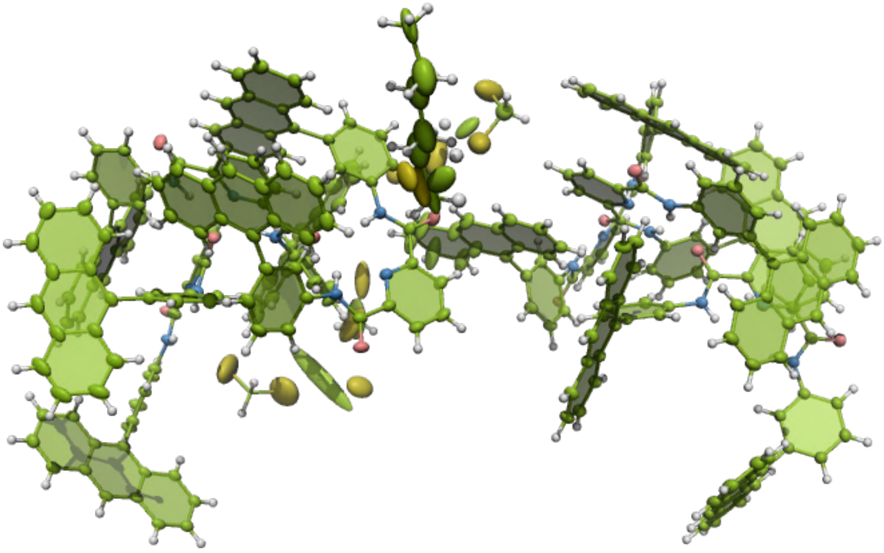

Supplementary Figure 20. The ORTEP drawing structures of (**1**)$_6$. The thermal ellipsoids are drawn at 30% possibility. Green: carbon, blue: nitrogen, yellow: chlorine, and pink: oxygen.

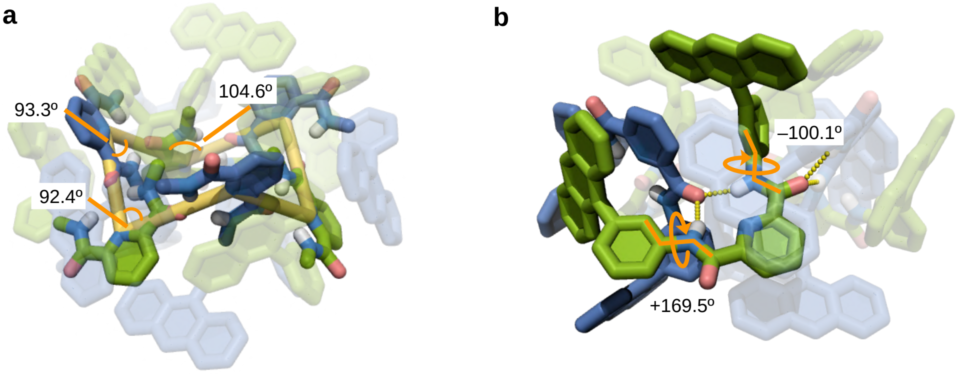

Supplementary Figure 21. Two side views of (**1**)$_6$. (a) The highlight of zig-zag assembly (**1**)$_6$ and N-N-N angle. Each nitrogen atom of pyridine was connected with yellow stick. (b) Dihedral angles between amide and *m*-phenylene ring (C-N-C-C). Hydrogen atoms (except N*H*) were omitted for clarity.

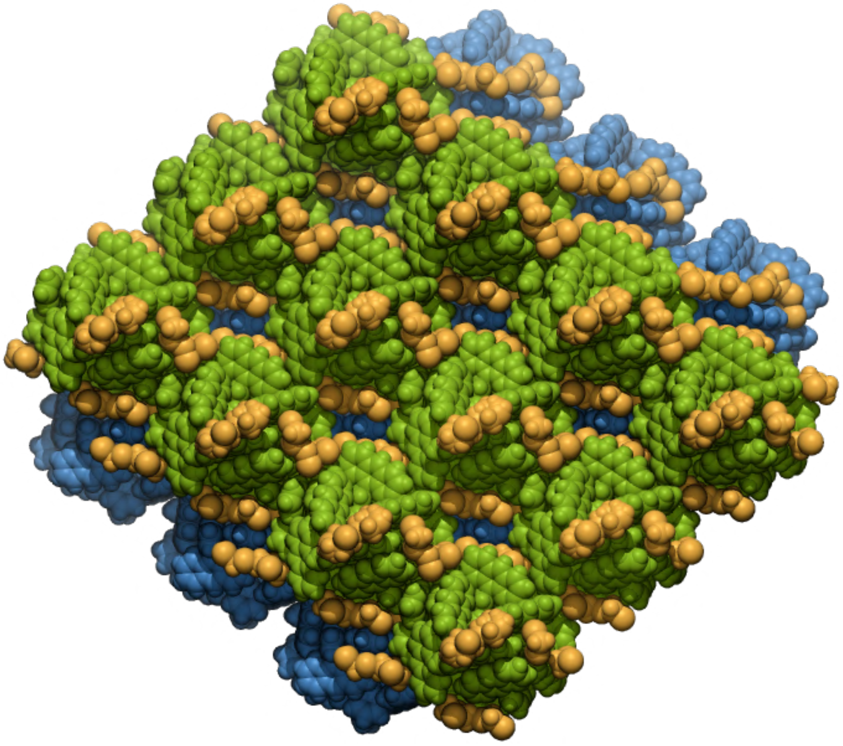

Supplementary Figure 22. CPK representation of packing structure. Each hexamer (**1**)$_6$ was shown in green and blue colors, depending on the layer. The disordered solvent molecules (*n*-hexane and CH_2Cl_2 with yellow color) are located in the groove of each hexamer.

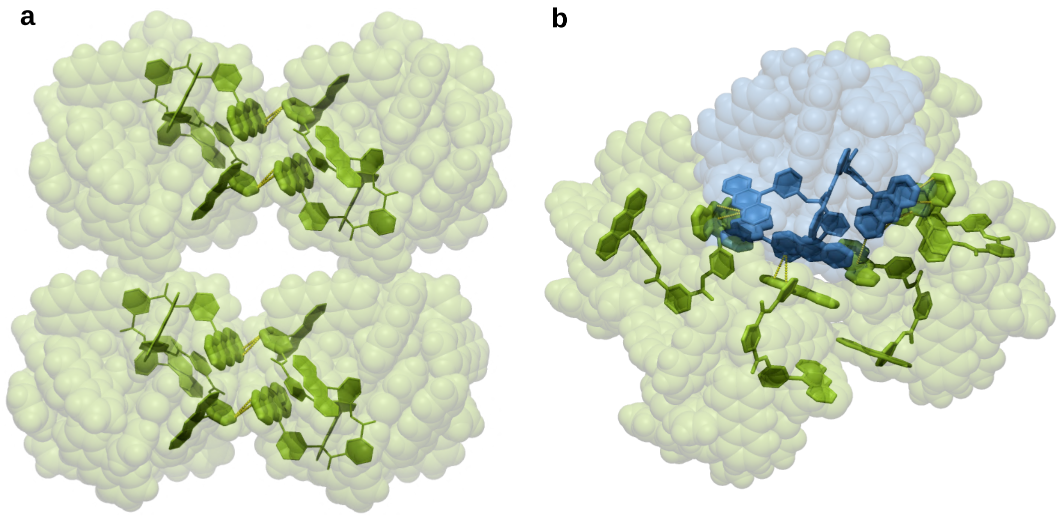

Supplementary Figure 23. Transparent CPK and stick representations of intermolecular interactions between each hexamer. (a) In the same layer and (b) with the different layer. The monomers showing inter-hexamer π-π and CH-π interactions are described in thick-stick model. Yellow dotted lines indicate the distances of less than 3.5 Å for C-C atom or C-centroid of the ring. Solvent molecules were omitted for clarity.

Supplementary Table 2. Crystal data and structure refinement for **1**.

Identification code	mANT	
Empirical formula	C47.99 H33.1 Cl1.25 N3 O2	
Formula weight	728.18	
Temperature	123 K	
Wavelength	1.54184 Å	
Crystal system	triclinic	
Space group	*P*-1	
Unit cell dimensions	a = 21.3875(2) Å	α = 98.9480°
	b = 21.4870(2) Å	β = 100.701(1)°
	c = 25.2973(1) Å	γ = 95.4240°
Volume	11192.69(16) $Å^3$	
Z	12	
Density (calculated)	1.296 Mg/m^3	
Absorption coefficient	1.424 mm^{-1}	
F(000)	4552.0	
Crystal size	0.209 × 0.138 × 0.101 mm^3	
2 Theta range for data collection	6.334 to 149.006°.	
Index ranges	–26<=h<=26, –26<=k<=26, –31<=l<=31	
Reflections collected	160091	
Independent reflections	45288 [R(int) = 0.0357]	
Completeness to θ = 67.684°	98.9 %	
Absorption correction	Gaussian	
Refinement method	Full-matrix least-squares on F^2	
Data / restraints / parameters	45288 / 99 / 2997	
Goodness-of-fit on F^2	1.029	
Final R indices [I>2σ(I)]	R_1 = 0.0942, wR_2 = 0.2854	
R indices (all data)	R_1 = 0.1048, wR_2 = 0.2970	
Largest diff. peak and hole	1.316 and –1.765 e.$Å^{-3}$	

The supplementary crystallographic data (CCDC 2158897) can be obtained free of charge from the Cambridge Crystallographic Data Centre via www.ccdc.cam.ac.uk/data_request/cif.

1.6. A triple-layered macrocycle $(\mathbf{1})_6$

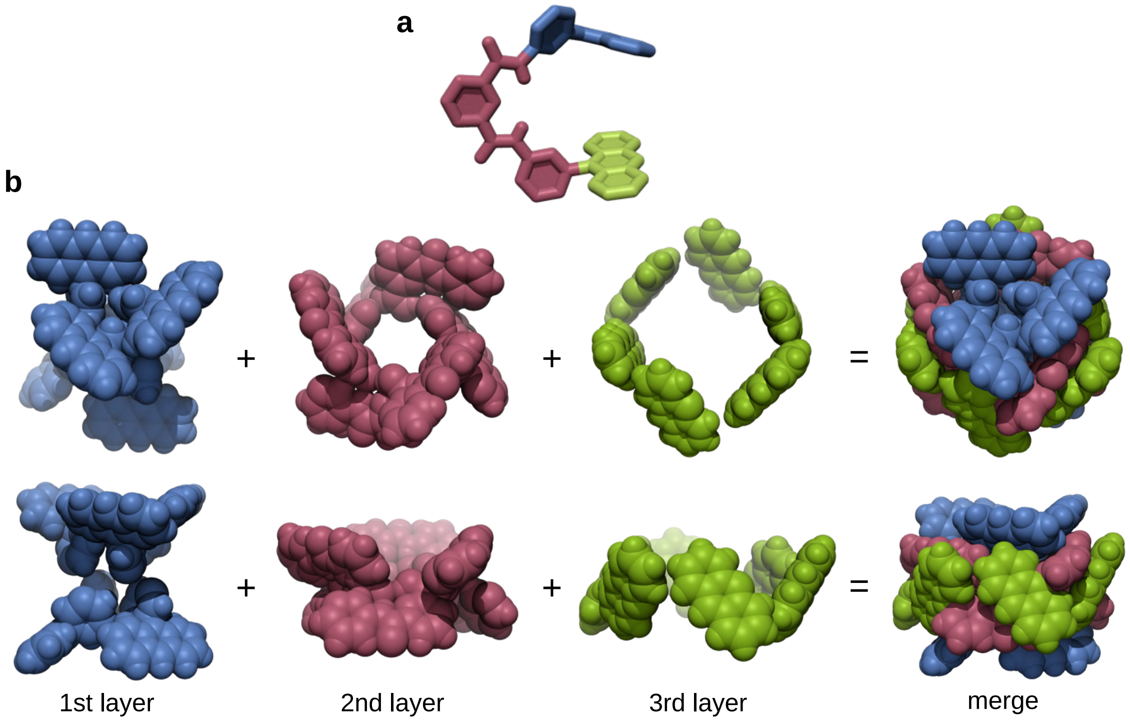

Supplementary Figure 24. Components of a triple-layered self-complementary hexamer. (a) A monomer in stick model with colors corresponding to each wall. Hydrogen atoms (except N*H*) were omitted for clarity. (b) Top view (top) and side view (bottom) of 1st, 2nd, and 3rd layers of the hexamer.

1.7. UV-vis and fluorescence spectra of $(\mathbf{1})_6$

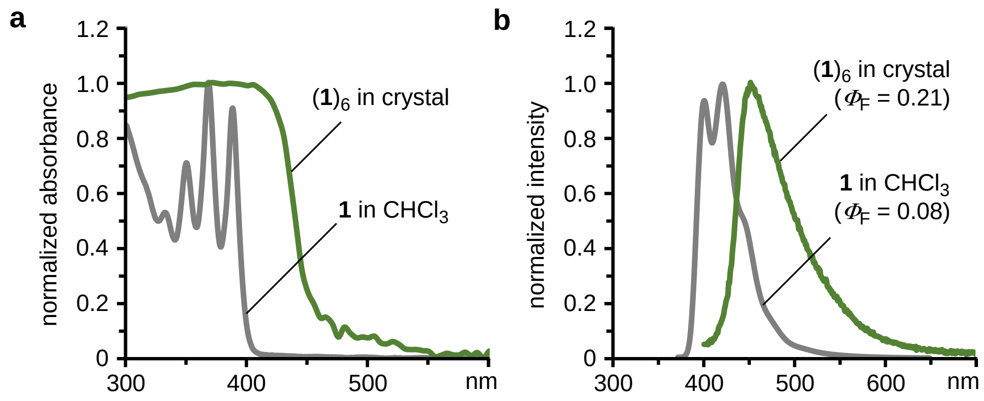

Supplementary Figure 25. Photophysical properties of **1** and $(\mathbf{1})_6$. (a) UV-vis and (b) fluorescence spectra with fluorescence quantum yields (Φ_F) of **1** in $CHCl_3$ (9 μM, λ_{ex} = 367 nm, 298 K) and $(\mathbf{1})_6$ in the solid state (λ_{ex} = 365 nm, 298 K).

1.8. Assembling process of (1)$_6$

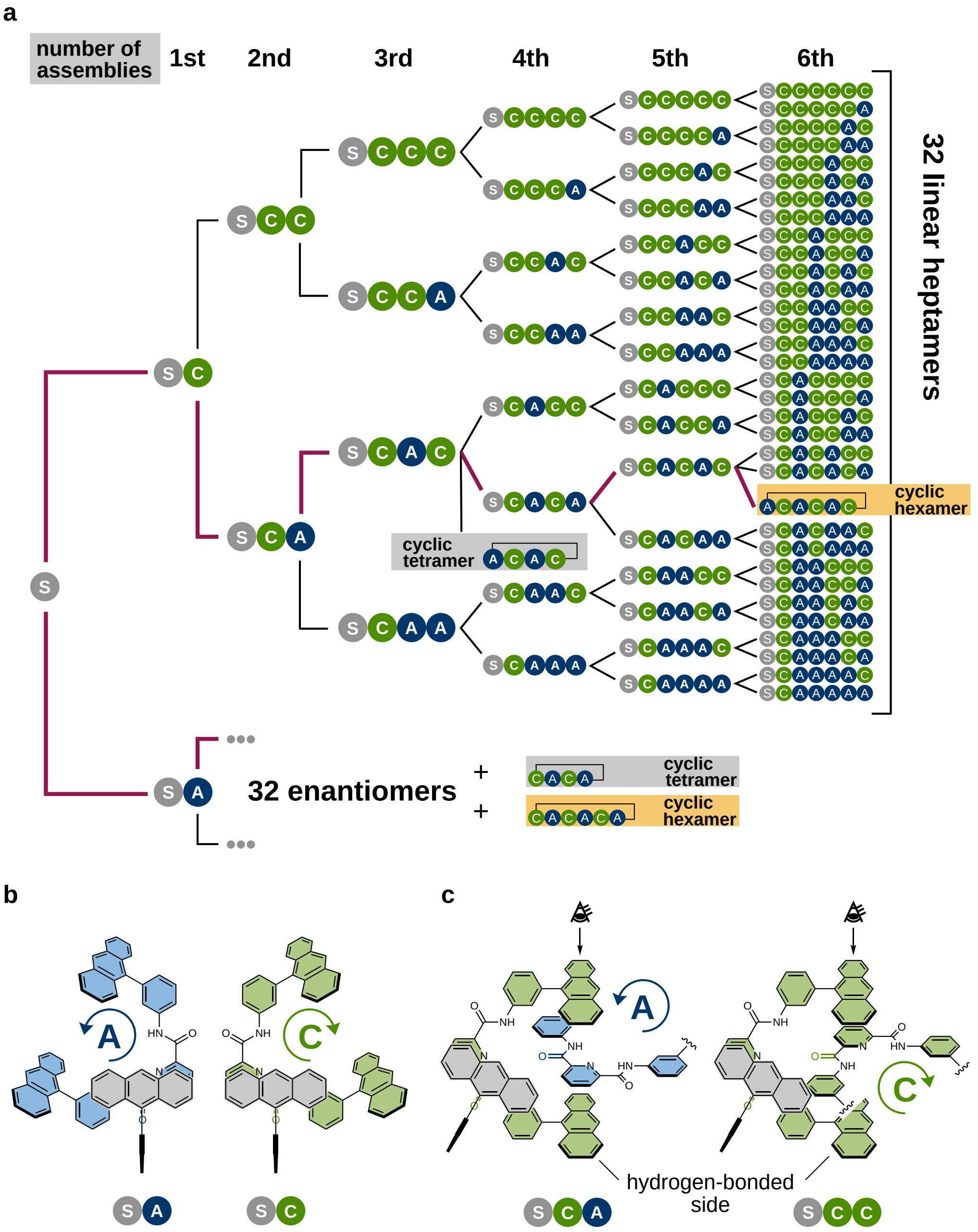

Supplementary Figure 26. Schematic representation of assembling process of (**1**)$_6$. (a) A tree diagram of possible isomers during six sets of self-complementary assemblies of **1**. 32 linear heptamers and a cyclic hexamer (and a cyclic tetramer) might be statistically formed with their enantiomers. Conformational structures of (b) SC and SA from 1st assembly and (c) SCA and SCC from 2nd assembly of SC isomer. “S” indicates an initial tweezers **1** of the self-complementary assembly. Looking from the anthracene front which is hydrogen-unbonded site, the bound other **1** pointed clockwise (C) and anti-clockwise (A) are colored green and blue, respectively.

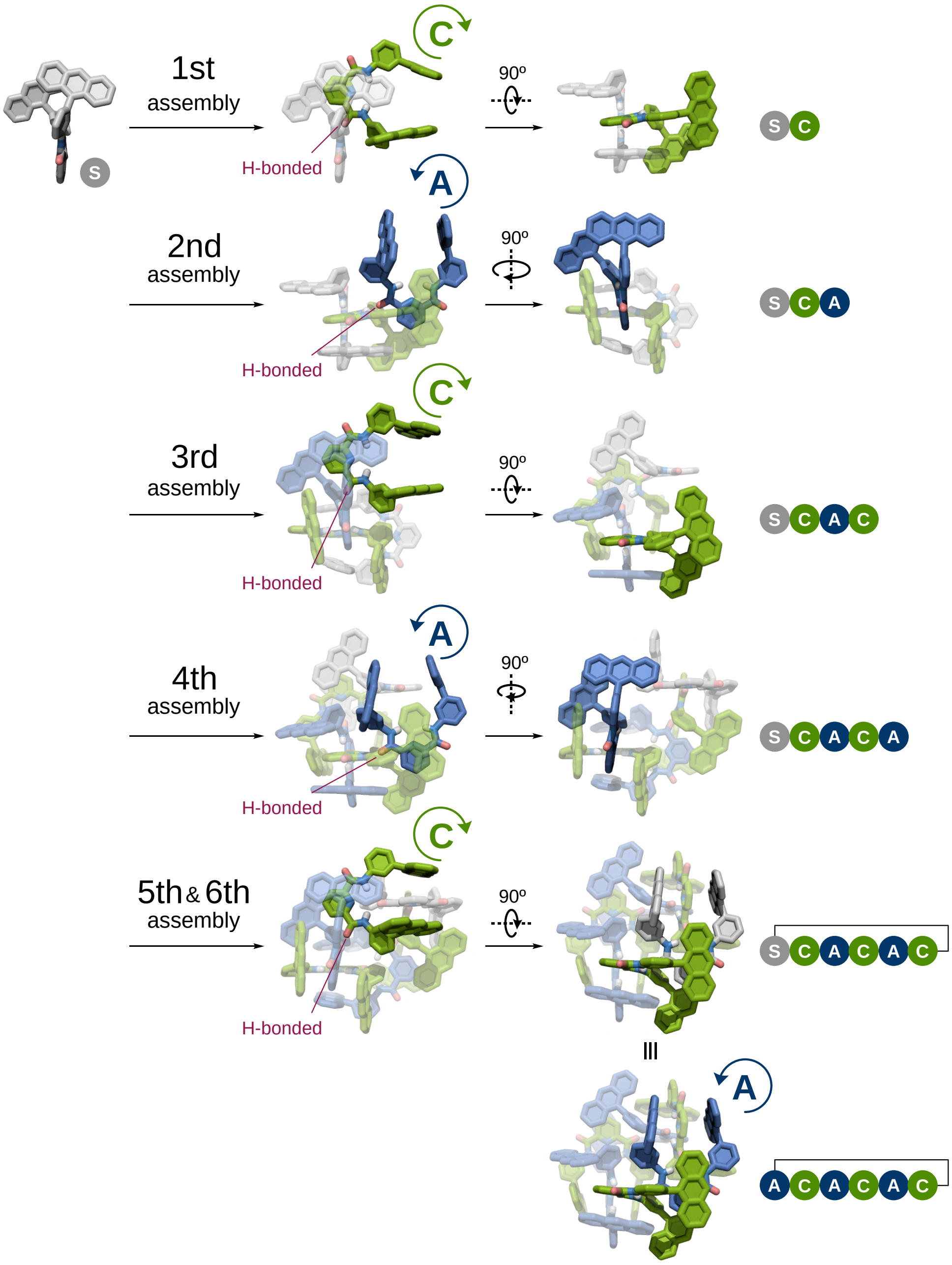

Supplementary Figure 27. Schematic representation of assembling process to cyclic hexamer (**1**)$_6$ as ACACAC assembly. Hydrogen atoms (except N*H*) were omitted for clarity.

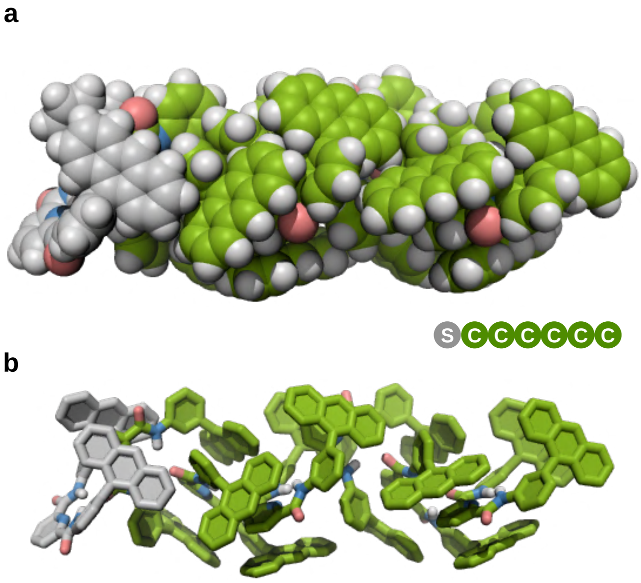

Supplementary Figure 28. Optimized structure of possible linear heptamer SCCCCCC calculated by molecular mechanics. (a) Space filling and (b) stick representations.

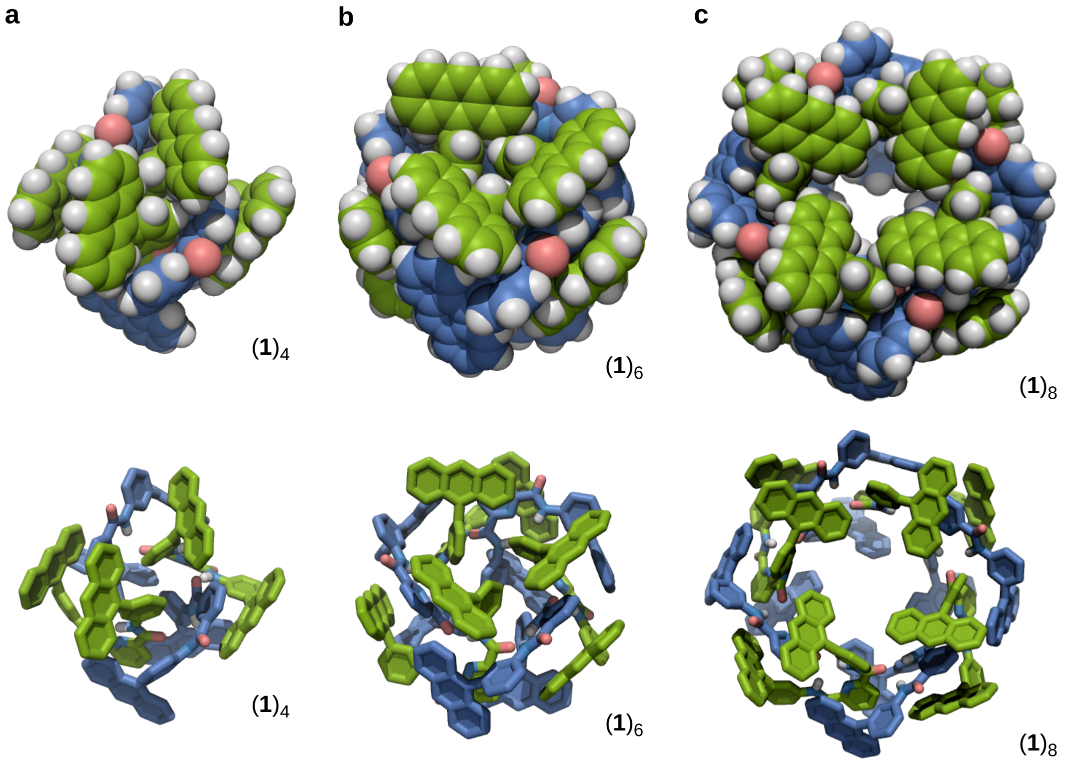

Supplementary Figure 29. Space filling (top) and stick (bottom) representations of possible cyclic isomers. A cyclic (a) tetramer (**1**)$_4$, (b) hexamer (**1**)$_6$ in this study, and (c) octamer (**1**)$_8$. Hydrogen atoms (except N*H*) are omitted for clarity in the stick representation. A cyclic tetramer (**1**)$_4$ and octamer (**1**)$_8$ were calculated by molecular mechanics.

1.9. Powder X-ray diffractions and IR measurement of (1)$_6$

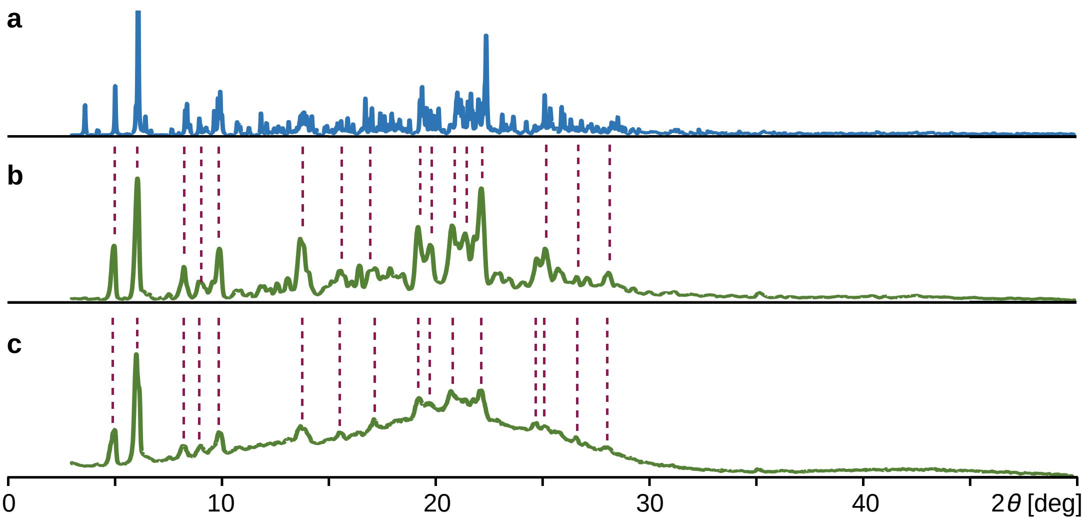

Supplementary Figure 30. Powder X-ray diffraction patterns at 298 K. (a) A simulation pattern based on the diffraction pattern of single-crystal X-ray analysis of (**1**)$_6$. Diffraction patterns of (b) cyclic hexamer (**1**)$_6$ and (c) the resultant solid after grinding by a mortar for 3 minutes. Intensities are shown as arbitrary units.

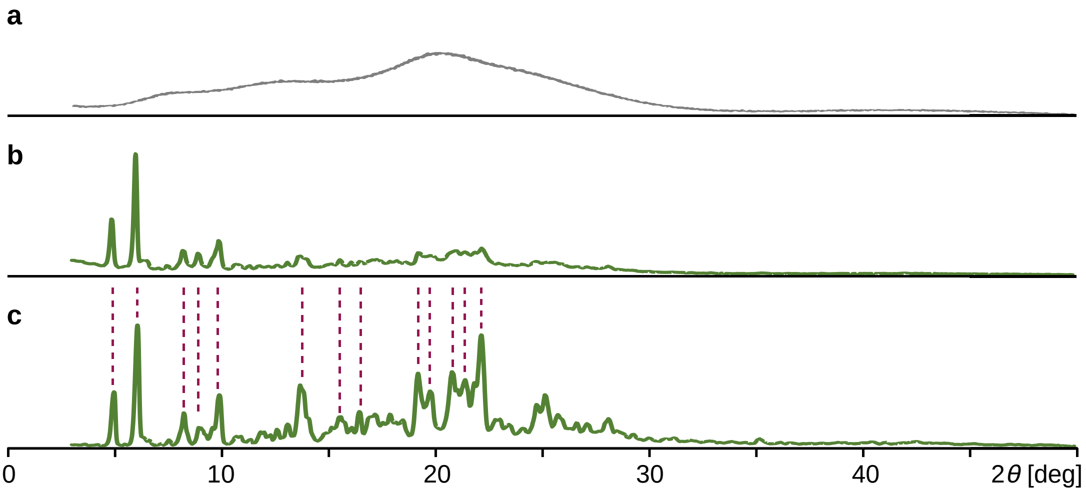

Supplementary Figure 31. Powder X-ray diffraction patterns at 298 K. The resultant bulk powders of **1** after (a) heating at 200 ºC and then (b) exposure to CH_2Cl_2 vapor (Note: a 1.5 mL glass tube containing crystalline powders of (**1**)$_6$ (ca. 30 mg) was heated at 200 ºC for 30 min. The melted compound was cooled and scratched with a spatula to be powder form. Afterward, the glass tube was placed in a 30 mL glass vial with CH_2Cl_2 (5 mL) and exposed the solid to CH_2Cl_2 vapor in the sealed vial for 2 h at room temperature.). (c) A diffraction pattern of (**1**)$_6$. Intensities are shown as arbitrary units.

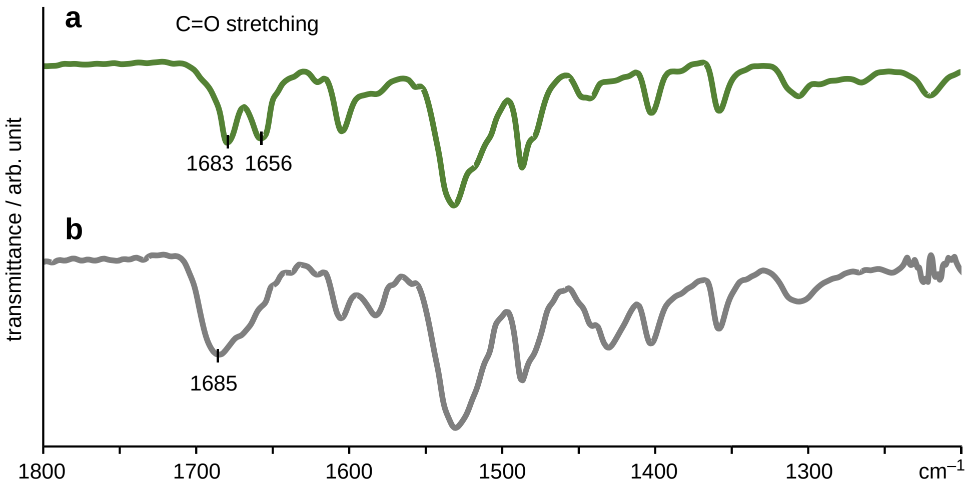

Supplementary Figure 32. ATR-FTIR spectra of **1**. (a) The crystalline powder of (**1**)$_6$ and (b) the amorphous powder of **1**.

1.10. Single crystal X-ray diffraction of $(2)_n$ and $(3)_n$

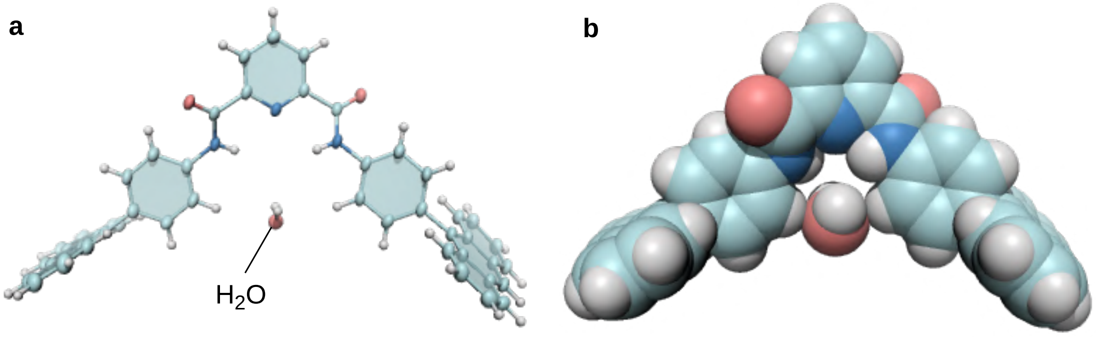

Supplementary Figure 33. Representation of X-ray crystal structure of **2**. (a) The ORTEP drawing structures of **2**. The thermal ellipsoids are drawn at 50% possibility and its (b) CPK representation. Light blue: carbon, blue: nitrogen, and pink: oxygen.

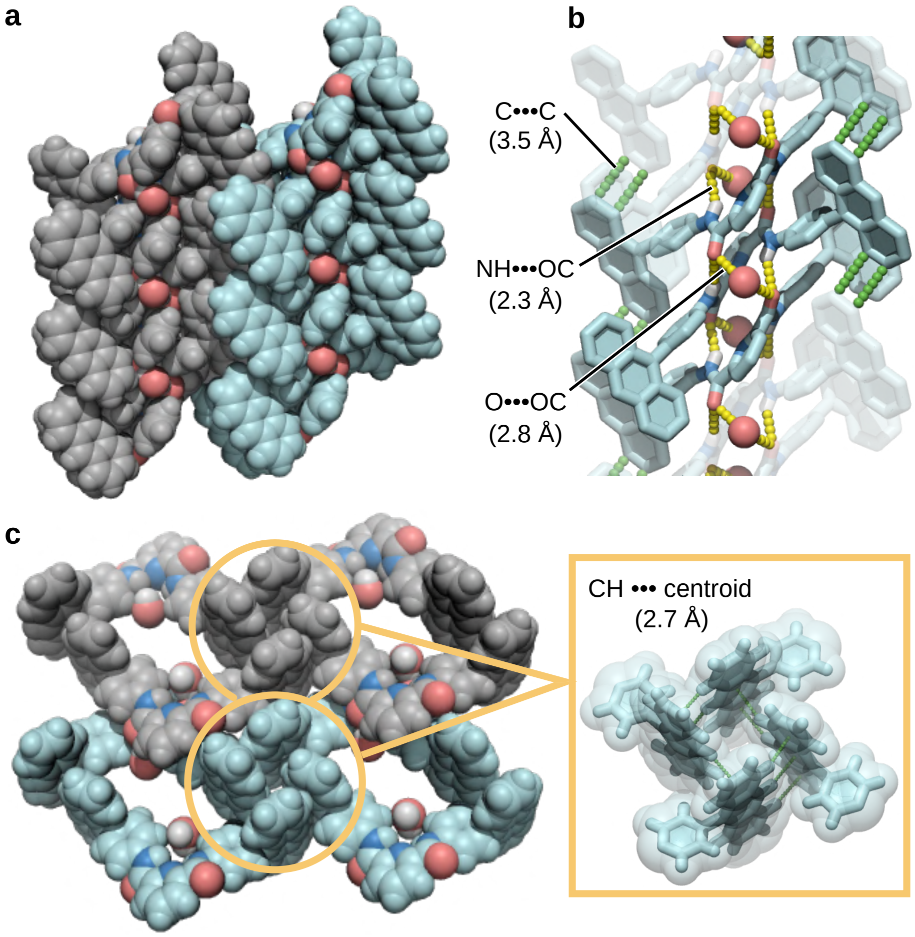

Supplementary Figure 34. Representation of packing structure of **2**. (a) CPK model, (b) showing intermolecular NH-OC (yellow dotted line) and OH-OC hydrogen-bondings (yellow dotted line) and π-π interactions (green dotted line), and (c) intermolecular CH-π interactions between CH and centroid of ring (green dotted line). The average distances are written in parentheses.

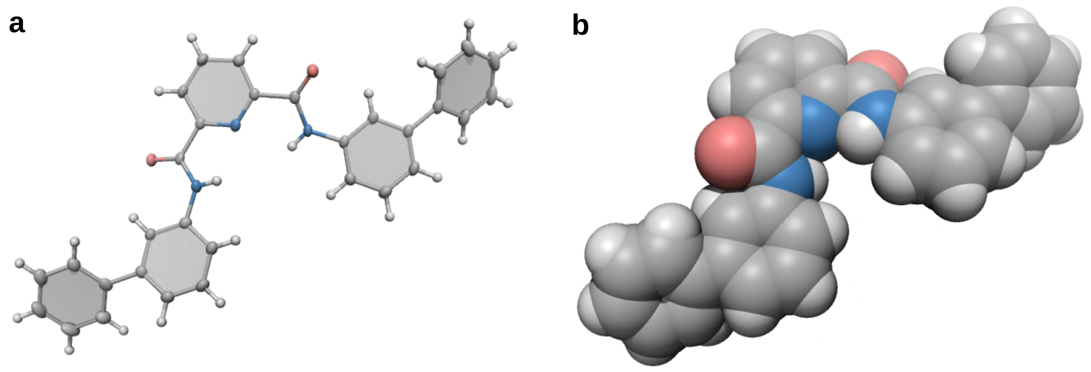

Supplementary Figure 35. Representation of X-ray crystal structure of **3**. (a) The ORTEP drawing structures of **3**. The thermal ellipsoids are drawn at 50% possibility and its (b) CPK representation. Gray: carbon, blue: nitrogen, and pink: oxygen.

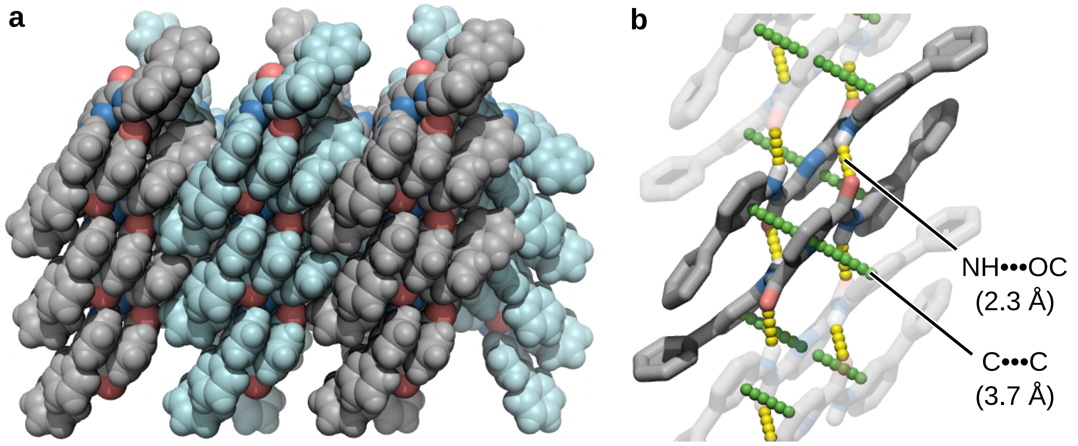

Supplementary Figure 36. Representation of packing structure of **3**. (a) CPK model, (b) showing intermolecular NH-OC hydrogen-bondings (yellow dotted line) and intermolecular CH-π interactions between CH and centroid of ring (green dotted line). The average distances are written in parentheses.

Supplementary Table 3. Crystal data and structure refinement for **2**.

Identification code	pANT	
Empirical formula	C47 H31.65 N3 O2.19	
Formula weight	673.44	
Temperature	123 K	
Wavelength	1.54184 Å	
Crystal system	triclinic	
Space group	*P*-1	
Unit cell dimensions	a = 8.6339(2) Å	α = 94.037(3)°
	b = 13.4549(5) Å	β = 99.520(2)°
	c = 15.8267(4) Å	γ = 107.573(3)°
Volume	1714.40(9) $Å^3$	
Z	2	
Density (calculated)	1.304 Mg/m^3	
Absorption coefficient	0.635 mm^{-1}	
F(000)	704.0	
Crystal size	0.17 × 0.03 × 0.03 mm^3	
2 Theta range for data collection	5.69 to 145.580°.	
Index ranges	–10<=h<=8, –16<=k<=16, –19<=l<=19	
Reflections collected	17343	
Independent reflections	6268 [R(int) = 0.0384]	
Completeness to θ = 68.13°	99.60 %	
Absorption correction	Gaussian	
Refinement method	Full-matrix least-squares on F^2	
Data / restraints / parameters	6268 / 0 / 479	
Goodness-of-fit on F^2	1.112	
Final R indices [I>2σ(I)]	R_1 = 0.0532, wR_2 = 0.1190	
R indices (all data)	R_1 = 0.0725, wR_2 = 0.1271	
Largest diff. peak and hole	0.211 and –191 e.$Å^{-3}$	

The supplementary crystallographic data (CCDC 2158909) can be obtained free of charge from the Cambridge Crystallographic Data Centre via www.ccdc.cam.ac.uk/data_request/cif.

Supplementary Table 4. Crystal data and structure refinement for **3**.

Identification code	mPh	
Empirical formula	C31 H23 N3 O2	
Formula weight	469.52	
Temperature	123 K	
Wavelength	1.54184 Å	
Crystal system	monoclinic	
Space group	$P\ 2_1/n$	
Unit cell dimensions	a = 8.5846(2) Å	α = 90°
	b = 13.5214(4) Å	β = 98.635(2)°
	c = 20.5679(5) Å	γ = 90°
Volume	2360.37(11) $Å^3$	
Z	4	
Density (calculated)	1.321 Mg/m^3	
Absorption coefficient	0.666 mm^{-1}	
F(000)	984.0	
Crystal size	0.1 × 0.1 × 0.1 mm^3	
2 Theta range for data collection	7.852 to 152.846°.	
Index ranges	–10<=h<=7, –16<=k<=13, –25<=l<=25	
Reflections collected	16780	
Independent reflections	4566 [R(int) = 0.0384]	
Completeness to θ = 67.97°	99.91 %	
Absorption correction	multi-scan	
Refinement method	Full-matrix least-squares on F^2	
Data / restraints / parameters	4566 / 0 / 325	
Goodness-of-fit on F^2	1.056	
Final R indices [I>2σ (I)]	R_1 = 0.0997, wR_2 = 0.2891	
R indices (all data)	R_1 = 0.1036, wR_2 = 0.2919	
Largest diff. peak and hole	0.850 and –0.378 e.$Å^{-3}$	

The supplementary crystallographic data (CCDC 2158891) can be obtained free of charge from the Cambridge Crystallographic Data Centre via www.ccdc.cam.ac.uk/data_request/cif.

1.11. Thermal analysis

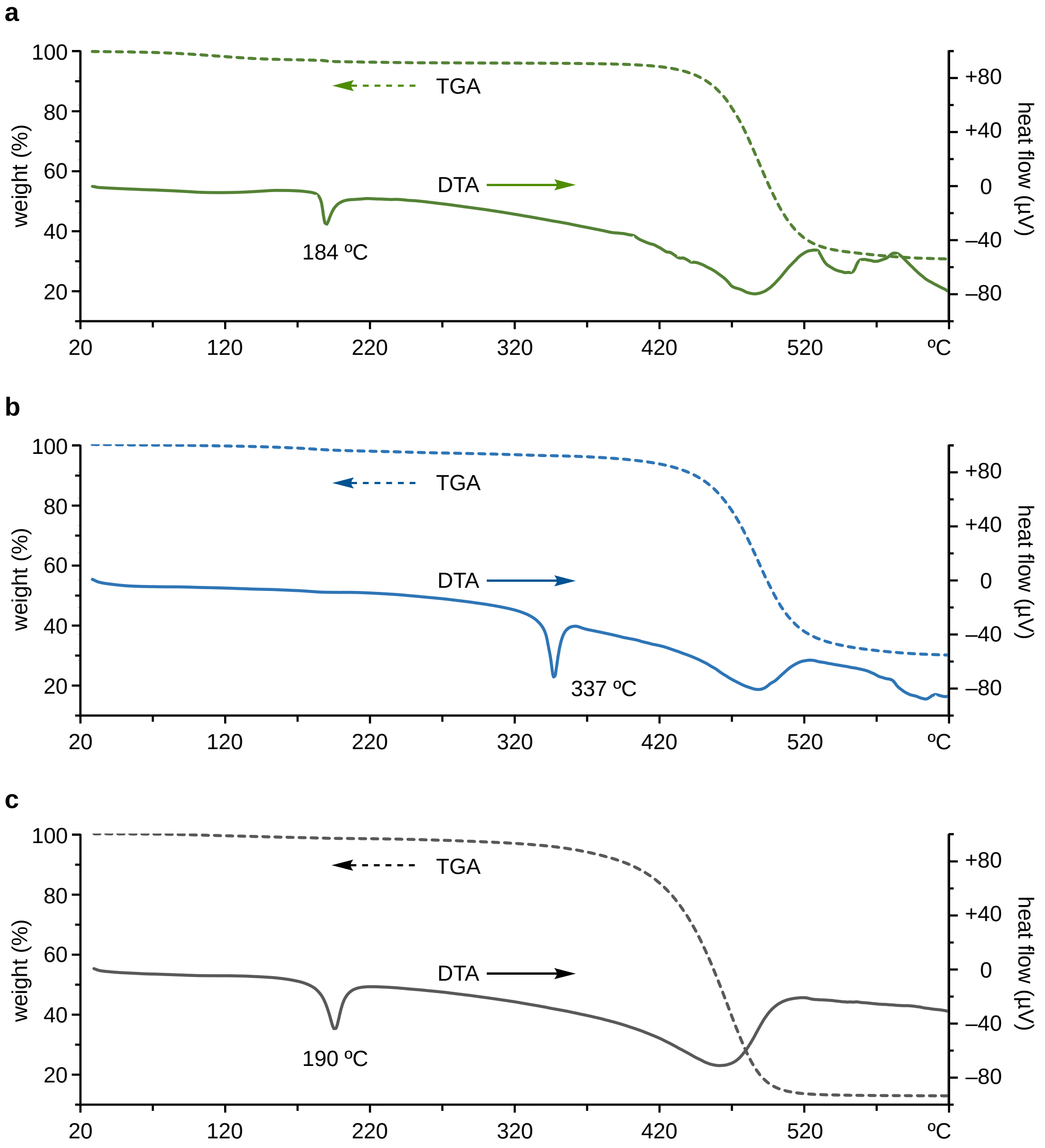

Supplementary Figure 37. TG-DTA curves (heating rate of 10 ºC/min from 30 ºC to 600 ºC). (a) (**1**)$_6$, (b) (**2**)$_n$, and (c) (**3**)$_n$ (TGA: dash line, DTA: solid line).

1.12. Single crystal X-ray diffraction of $(\mathbf{1})_6$•$(TFA)_2$

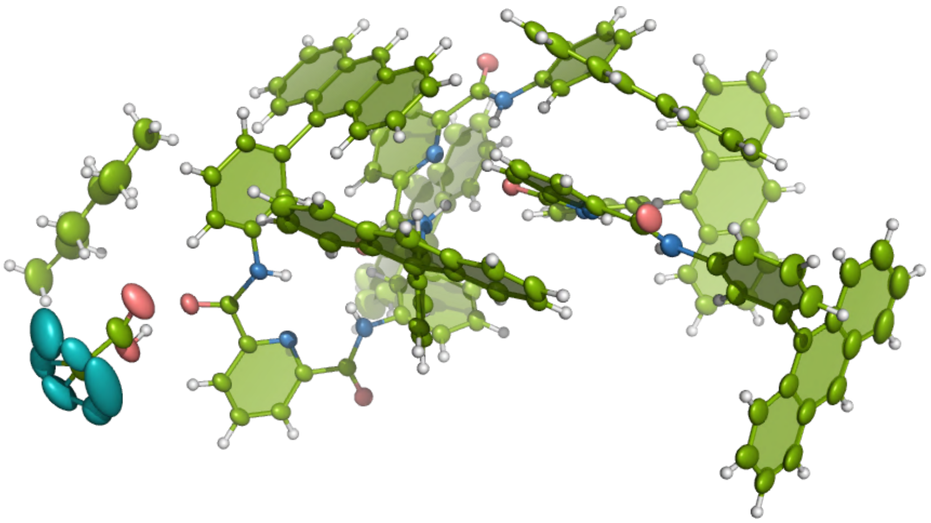

Supplementary Figure 38. The ORTEP drawing structures of $(\mathbf{1})_3$•(TFA). The thermal ellipsoids are drawn at 30% possibility. Green: carbon, blue: nitrogen, bluish green: fluorine, and pink: oxygen.

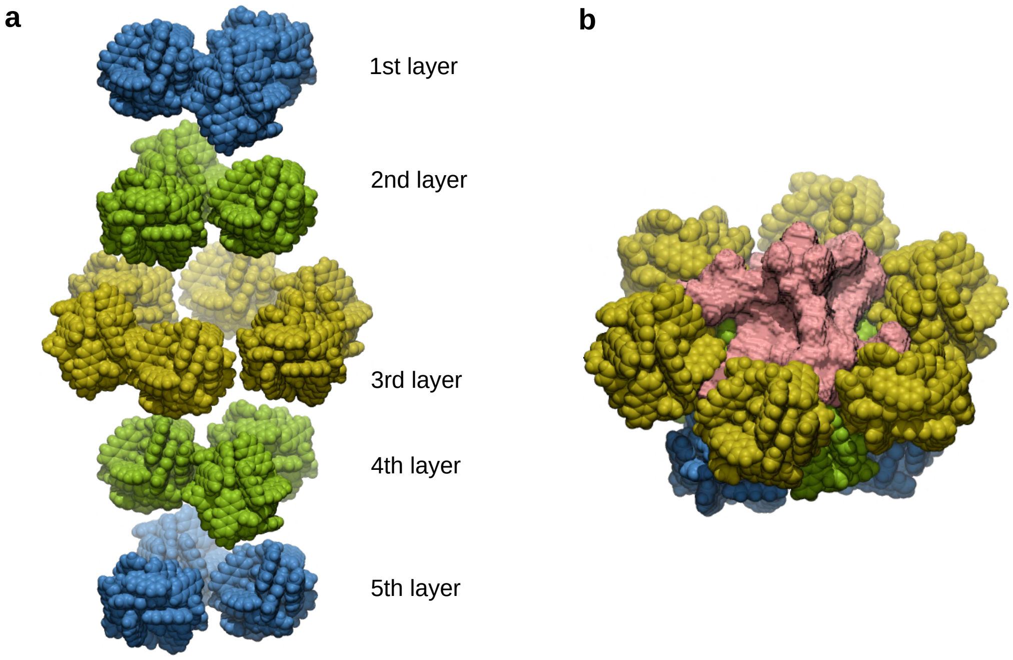

Supplementary Figure 39. Structural details of $[(\mathbf{1})_6]_{18}$. (a) An exploded view of $[(\mathbf{1})_6]_{18}$. (b) A cross-section diagram of $[(\mathbf{1})_6]_{18}$ with cavity (pink color).

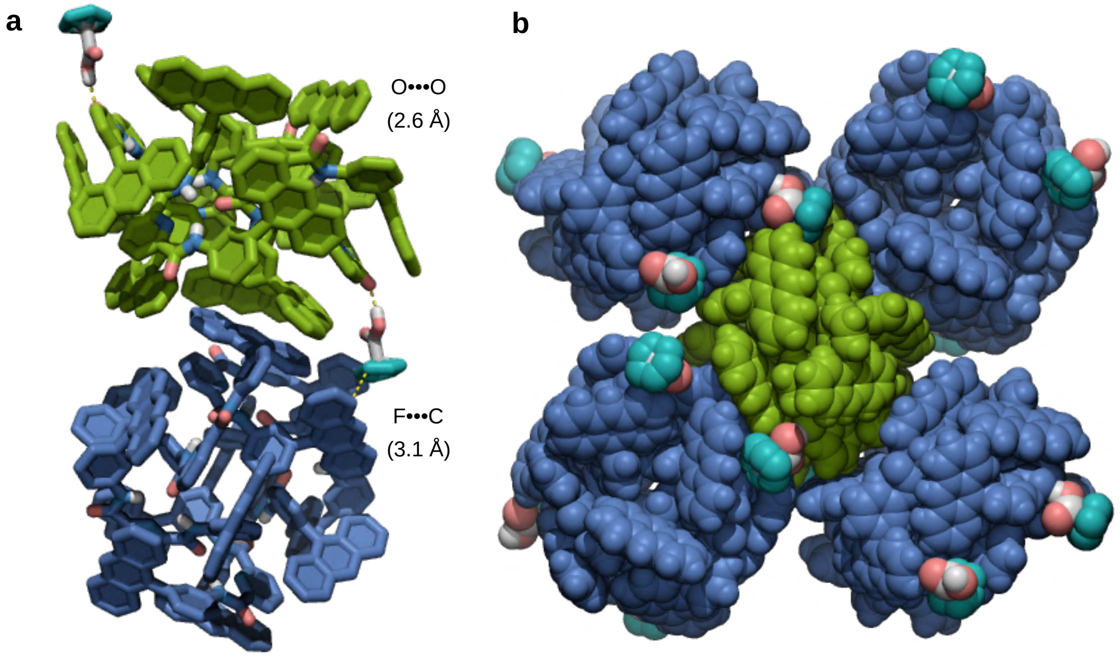

Supplementary Figure 40. The role of TFA molecules. (a) Position of TFA molecules in the crystal. (b) Intermolecular hydrogen-bonds and halogen-π interactions between (**1**)$_6$ and TFA. *n*-Hexane molecules are omitted for clarity.

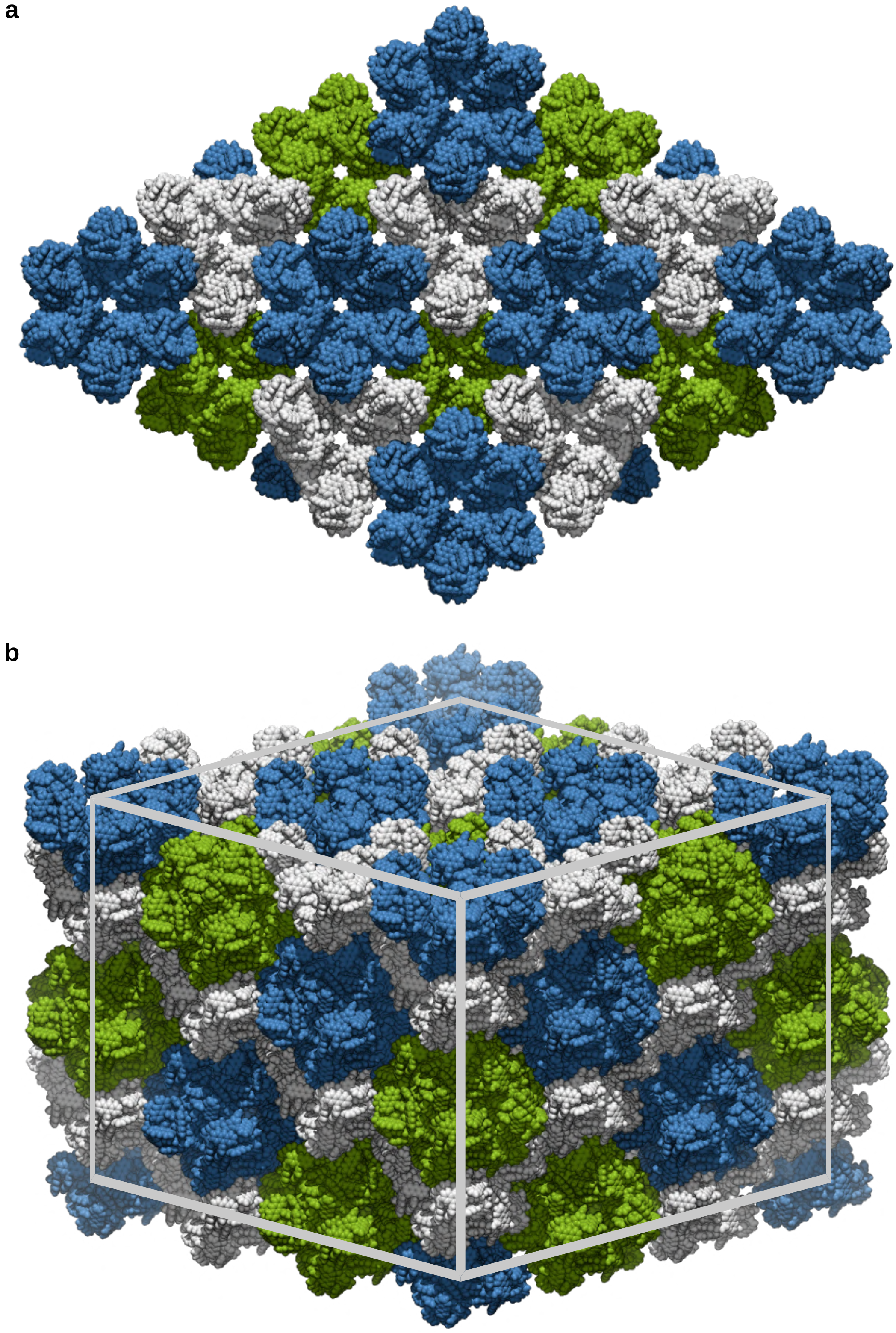

Supplementary Figure 41. CPK representation of crystal packing of (**1**)$_6$•(TFA)$_2$. (a) Top view and (b) side view. Each hexamer [(**1**)$_6$]$_{18}$ was shown in green and blue colors, and grid hexamers (**1**)$_6$ were colored gray. The solvent and TFA molecules are omitted for clarity.

a

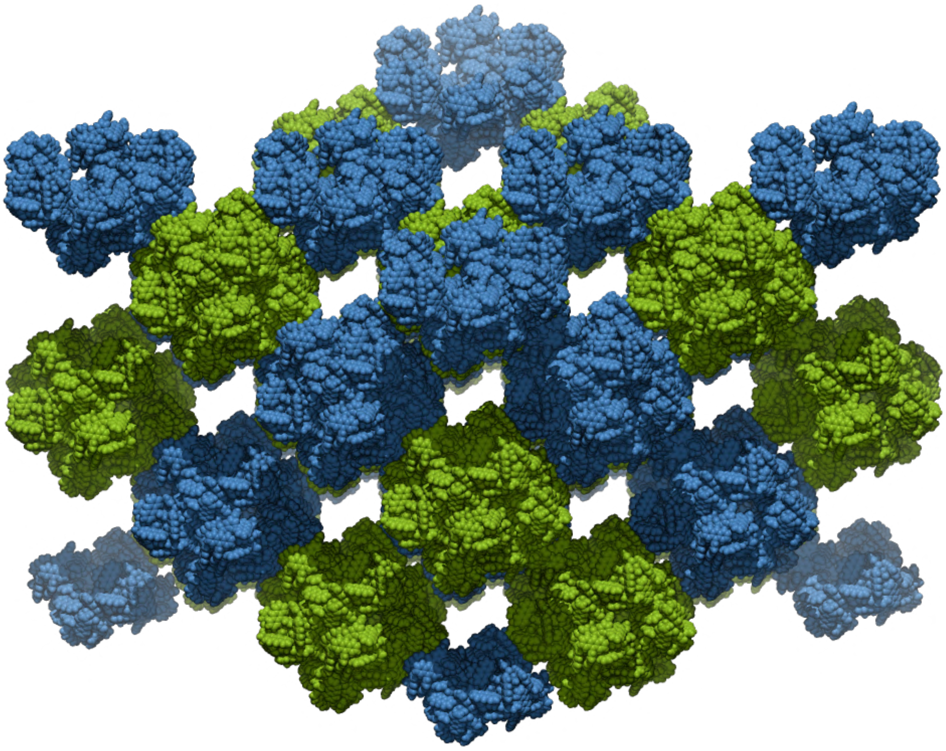

b

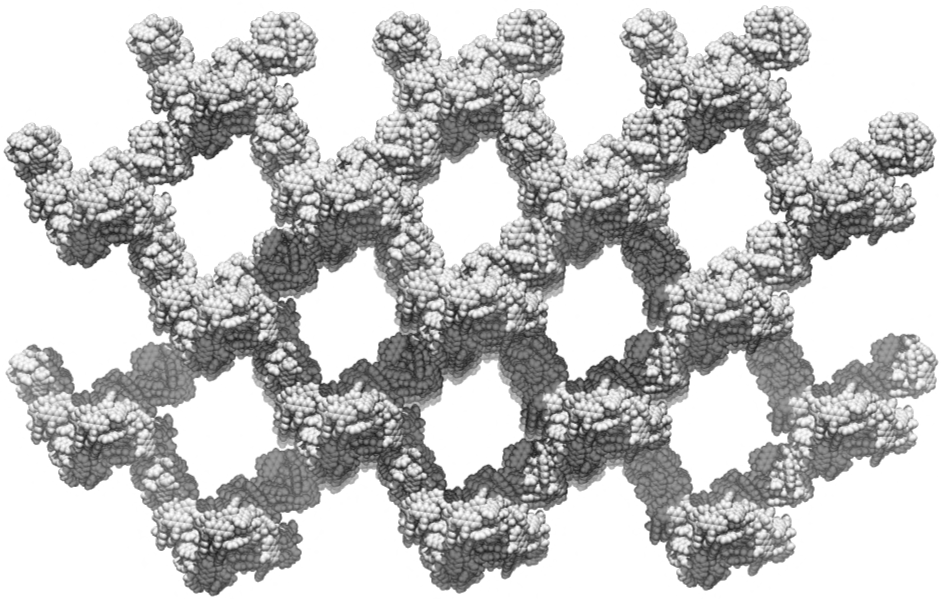

Supplementary Figure 42. Extracted crystal packing of (**1**)$_6$•(TFA)$_2$. (a) [(**1**)$_6$]$_{18}$ and (b) grid hexamer (**1**)$_6$. The solvent and TFA molecules are omitted for clarity.

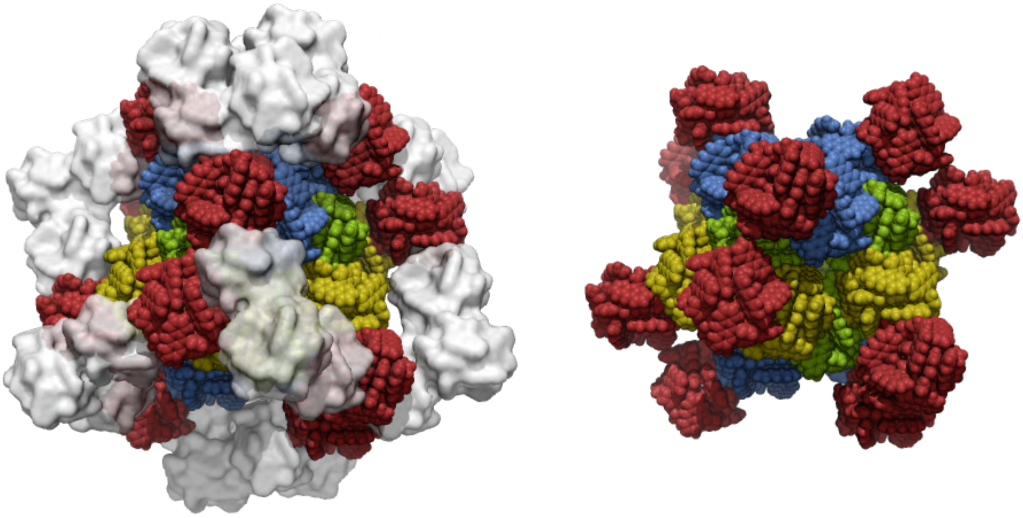

Supplementary Figure 43. Intermolecular interaction between $[(\mathbf{1})_6]_{18}$ and grid hexamer $(\mathbf{1})_6$. Interacting grid hexamers $(\mathbf{1})_6$ were colored in red.

Supplementary Table 5. Crystal data and structure refinement for (**1**)$_6$•(TFA)$_2$.

Identification code	mANT_TFA	
Empirical formula	C149 H108 F3 N9 O8	
Formula weight	2209.44	
Temperature	123 K	
Wavelength	1.54184 Å	
Crystal system	trigonal	
Space group	*R-3*	
Unit cell dimensions	a = 50.1889(8) Å	α = 90°
	b = 50.1889(8) Å	β = 90°
	c = 29.5529(4) Å	γ = 120°
Volume	64468(2) Å^3	
Z	18	
Density (calculated)	1.024 Mg/m^3	
Absorption coefficient	0.529 mm^{-1}	
F(000)	20808.0	
Crystal size	0.176 × 0.119 × 0.115 mm^3	
2 Theta range for data collection	5.048 to 152.04°.	
Index ranges	–45<=h<=61, –61<=k<=62, –36<=l<=36	
Reflections collected	86118	
Independent reflections	28430 [R(int) = 0.0441]	
Completeness to θ = 66.97°	99.29 %	
Absorption correction	multi-scan	
Refinement method	Full-matrix least-squares on F^2	
Data / restraints / parameters	28430 / 324 / 1553	
Goodness-of-fit on F^2	1.224	
Final R indices [I>2σ (I)]	R_1 = 0.0949, wR_2 = 0.3039	
R indices (all data)	R_1 = 0.1118, wR_2 = 0.3199	
Largest diff. peak and hole	0.773 and –0.482 e.Å^{-3}	

The supplementary crystallographic data (CCDC 2158899) can be obtained free of charge from the Cambridge Crystallographic Data Centre via www.ccdc.cam.ac.uk/data_request/cif.

Note: The acids and solvents within the lattice were significantly disordered and despite numerous attempts at modelling, including with rigid bodies no satisfactory model for the electron-density associated with them could be found. Therefore, the solvent mask function of OLex2 was employed to account for the highly disordered solvents and TFA. The diffuse solvent and additive molecules could not be conclusively assigned to CH_2Cl_2, *n*-hexane or TFA were therefore not included in the formula.

2. Supplementary Methods

2.1. General procedure for the synthesis of aminophenyl precursors

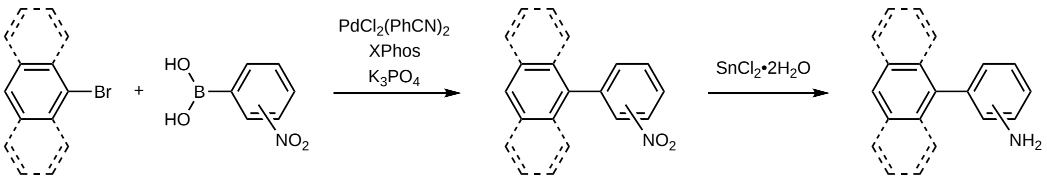

Aryl bromide (1.0 eq.), nitrophenylboronic acid (1.1 eq.), $PdCl_2(PhCN)_2$ (1.0 mol%), XPhos (2.0 mol%), and K_3PO_4 (3.0 eq.), degassed-THF/H_2O (300/1) were added to a two-neck flask under N_2 atmosphere. After striding at 70 ºC for 12 h, the resultant mixture was concentrated under reduce pressure. The crude product was purified by silica-gel short column chromatography (CH_2Cl_2) to give nitrophenyl compound. The obtained nitrophenyl compound (1.0 eq.), $SnCl_2$•$2H_2O$ (3.0 eq.), and THF were added to a 100 mL round bottom flask. After stirring at 70 ºC for 12 h, the resultant solution was diluted with AcOEt and washed with 10% NaOH aq.. The organic phase was dried over $MgSO_4$, filtrated, and concentrated under reduce pressure to afford aminophenyl precursor. 9-(3-Aminophenyl)anthracene (from 9-bromoanthracene and 3-nitrophenylboronic acid), 9-(4-aminophenyl)anthracene (from 9-bromoanthracene and 4-nitrophenylboronic acid), and 3-aminobiphenyl (from bromobenzene and 3-nitrophenylboronic acid) were synthesized in 94%, 90%, and 95% in two-step. ^{1}H NMR data were well consistent with the previous literatures.[1-3]

2.2. Synthesis of 1-3

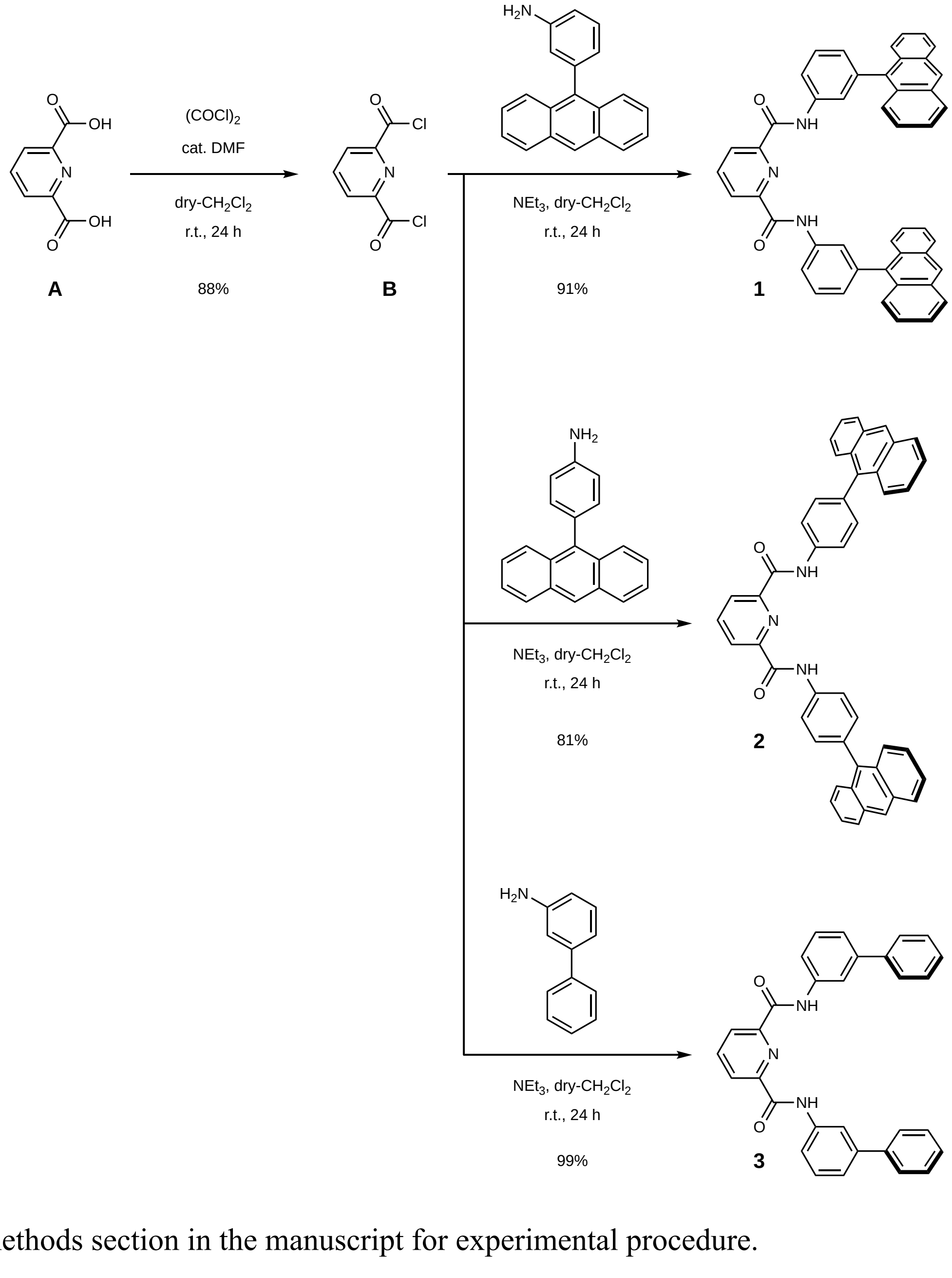

See methods section in the manuscript for experimental procedure.

2.3. Formation of self-complementary dimer

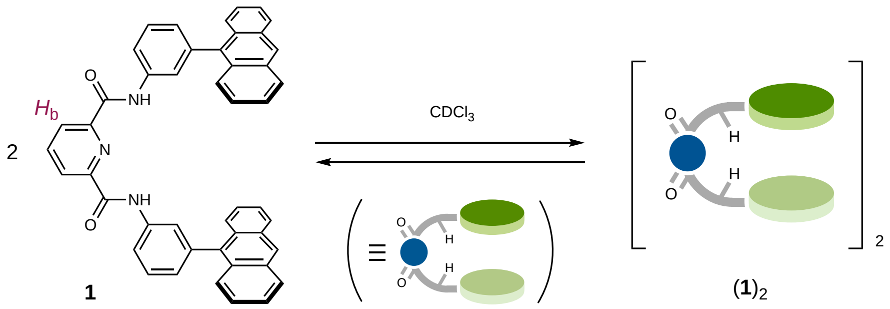

Molecular tweezers **1** was dissolved in $CDCl_3$ at 293 K. The spontaneous formation of self-complementary dimer (**1**)$_2$ was confirmed by NMR analysis.

2.4. Formation of self-complementary macrocycle

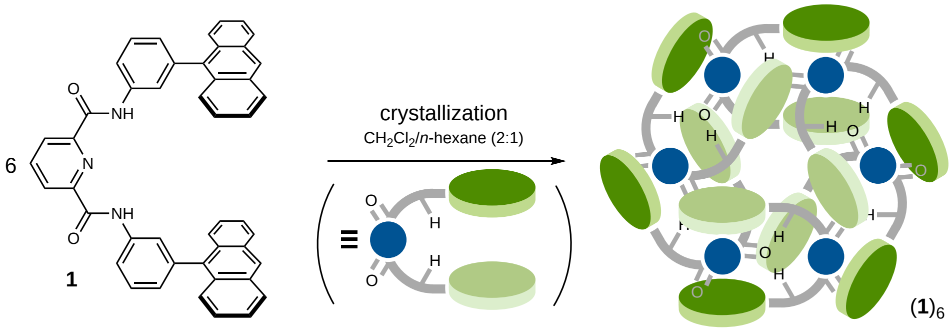

See methods section in the manuscript for experimental procedure.

2.5. Formation of hierarchical assemblies based on (1)$_6$

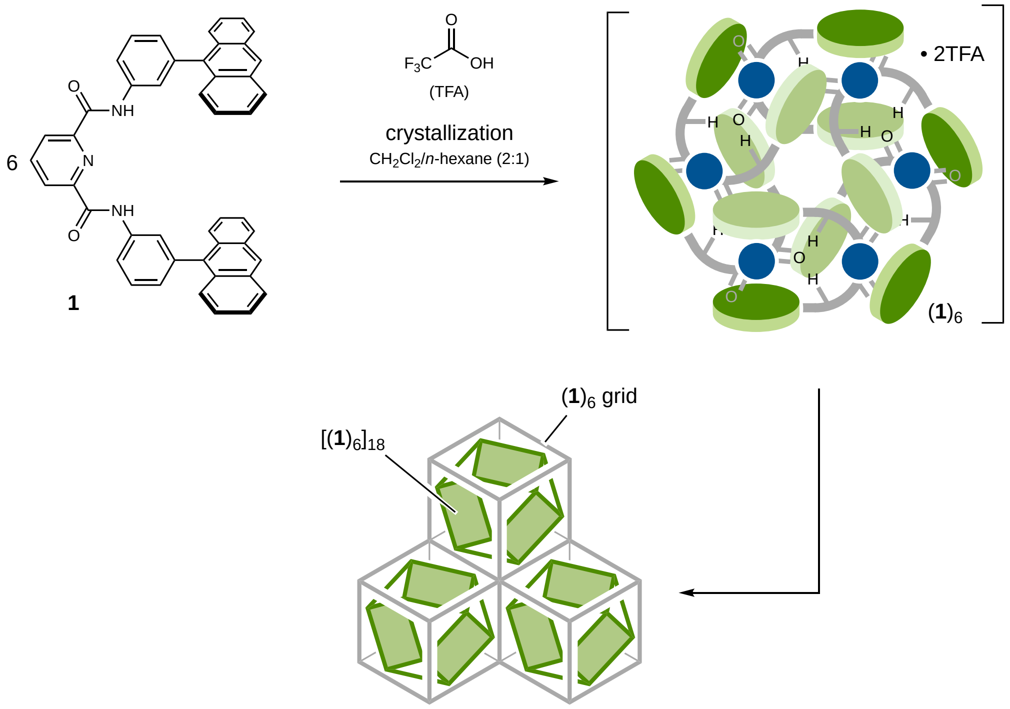

See methods section in the manuscript for experimental procedure.

3. Supplementary References

1. Li, Z., Ishizuka, H., Sei, Y., Akita, M. & Yoshizawa, M. Extended fluorochromism of anthracene trimers with a meta-substituted triphenylamine or triphenylphosphine core. *Chem. Asian J.* **7**, 1789–1794 (2012).
2. Liu, X., Dai, P., Gu, T., Wu, Q., Wei, H., Liu, S., Zhang, K. Y. & Zhao Q. Cyclometalated iridium(III) complexes containing an anthracene unit for sensing and imaging singlet oxygen in cellular mitochondria. *J. Inorg. Biochem.* **209**, 111106 (2020).
3. Li, Z., Gelbaum, C., Heaner, W. L. IV, Fisk, J., Jaganathan, A., Holden, B., Pollet, P. & Liotta, C. L. Palladium-catalyzed suzuki reactions in water with no added ligand: effects of reaction scale, temperature, pH of aqueous phase, and substrate structure. *Org. Process Res. Dev.* **20**, 1489−1499 (2016).
4. Iwanaga, T., Miyamoto, K., Tahara, K., Inukai, K., Okuhata, S., Tobe, Y. & Toyota, S. Chemistry of anthracene–acetylene oligomers XX: synthesis, structures, and self-association of anthracene–anthraquinone cyclic compounds with ethynylene linkers. *Chem. Asian J.* **7**, 935−943 (2012).
